# Single-nucleus transcriptomics reveals sepsis-related neurovascular dysfunction in the human hippocampus

**DOI:** 10.3389/fimmu.2025.1648278

**Published:** 2025-09-15

**Authors:** Liu Liu, Pengfei Li, Brent A. Wilkerson, Yan Wu, Meng Liu, Wei Jiang, Eric D. Hamlett, Steven L. Carroll, Hongkuan Fan

**Affiliations:** ^1^ Department of Pathology and Laboratory Medicine, Medical University of South Carolina, Charleston, SC, United States; ^2^ Department of Otolaryngology-Head and Neck Surgery, Medical University of South Carolina, Charleston, SC, United States; ^3^ Department of Psychiatry and Behavioral Sciences, Medical University of South Carolina, Charleston, SC, United States; ^4^ Department of Pharmacology and Immunology, Medical University of South Carolina, Charleston, SC, United States

**Keywords:** sepsis, neurovascular dysfunction, hippocampus, astrocytes, microglia, blood-brain barrier

## Abstract

**Introduction:**

Sepsis is increasingly recognized as a major precipitant of long-term cognitive impairment, yet the cellular mechanisms underlying hippocampal vulnerability remain elusive.

**Methods:**

We performed single-nucleus RNA sequencing of human hippocampal tissues from sepsis and control patients to profile neurovascular cell populations and their transcriptional changes.

**Results:**

We identified profound neurovascular alterations involving 21 distinct cell populations. Astrocytes and microglia exhibited marked polarization: Astrocyte 2 showed simultaneous upregulation of neurotoxic A1 and neuroprotective A2 gene signatures in sepsis, whereas Astrocyte 1 displayed reduced A1 activity and a relatively quiescent profile. Microglia 2 demonstrated a prominent M1-like inflammatory signature, including elevated HLA-DRA, IL1B, and TNF, while Microglia 1 downregulated both M1 and M2 markers, suggesting a hypo-responsive state. Intercellular communication analysis revealed intensified astrocyte–microglia interactions in the septic hippocampus. Endothelial and mural cells exhibited transcriptional signatures of blood-brain barrier disruption, oxidative stress, and compromised vascular homeostasis. Key molecular pathways associated with antigen presentation, cytokine signaling, and vascular permeability were selectively activated across neurovascular compartments.

**Discussion:**

These findings uncover a coordinated glial and vascular response to systemic inflammation, driven in part by dysfunctional astrocyte–microglia crosstalk and pro-inflammatory polarization. Such changes may underlie blood-brain barrier breakdown and contribute to sustained neuroinflammation and cognitive decline in sepsis survivors. Targeting glial-vascular signaling axes and modulating astrocyte or microglial polarization states may offer promising avenues for therapeutic intervention in post-sepsis neurological sequelae.

## 1 Introduction

Sepsis affects over 19 million people annually (1, 2) and despite declining mortality rates, the number of survivors continues to rise (3). Many of these individuals experience persistent neurological complications, including delirium, cognitive impairment, and psychiatric symptoms, collectively known as sepsis-associated encephalopathy (SAE) (3). SAE significantly contributes to long-term morbidity and mortality (4–6), yet its underlying mechanisms remain poorly understood, and targeted treatments are lacking.

Emerging evidence suggests that neurovascular injury and neuroinflammation, triggered by systemic metabolic disturbances and immune activation, play central roles in SAE (3, 4). Early phases of sepsis often involve cerebrovascular damage driven by hypotension, ischemia, and coagulopathy, which particularly affects vulnerable regions like the hippocampus (4, 7). This cerebrovascular damage might result in long-term neurological sequelae, particularly a decline in cognitive function (8). Within the brain, the neurovascular unit (NVU), composed of endothelial cells, pericytes, astrocytes, and neurons, regulates blood flow and immune responses (9). However, the contributions and interactions of NVU components and immune cells during sepsis remain largely unexplored.

Systemic inflammation during sepsis disrupts the blood-brain barrier (BBB), permitting peripheral cytokines and immune cells to infiltrate the brain and activate endothelial cells, pericytes, and microglia (10–13). This cascade amplifies neuroinflammation and promotes synaptic dysfunction (14). Proinflammatory cytokines, such as Interleukin-1 beta (IL-1β) and tumor necrosis factor-alpha (TNF-α), drive microglial polarization toward the M1 phenotype, characterized by the production of cytokines and reactive oxygen species that exacerbate neuronal injury (15–17). In contrast, M2-type microglia support anti-inflammatory responses and tissue repair (18). Similarly, astrocytes respond to microglial signals by adopting either a neurotoxic A1 phenotype, producing IL-1α, TNF-α, and Complement component 1q (C1q) (19), or a neuroprotective A2 phenotype that promotes neuronal survival and immune regulation (20). An imbalance favoring M1 microglia and A1 astrocytes is implicated in neurodegenerative and inflammatory conditions, including SAE, highlighting the critical role of glial phenotypic shifts in disease progression.

Recent single-cell and spatial transcriptomics studies in mouse models have begun to map the cellular landscapes and intercellular communication networks altered in SAE (21–23). These studies highlight the formation of pathological niches involving microglia, astrocytes, and vascular cells. However, insightful analyses in human brain tissue remain limited.

In this study, we analyzed *postmortem* hippocampal tissues from sepsis patients using single-nucleus RNA sequencing to characterize the cellular and molecular alterations in the NVU and immune compartments. We investigated dynamic changes in cell states and cell-cell communication, revealing a critical role for aberrant astrocyte–microglia interactions in driving neurovascular dysfunction in sepsis. These findings provide new insights into SAE pathogenesis and potential therapeutic targets.

## 2 Results

### 2.1 Sepsis-associated alterations to microglia and astrocyte clusters

**Figure 1:**
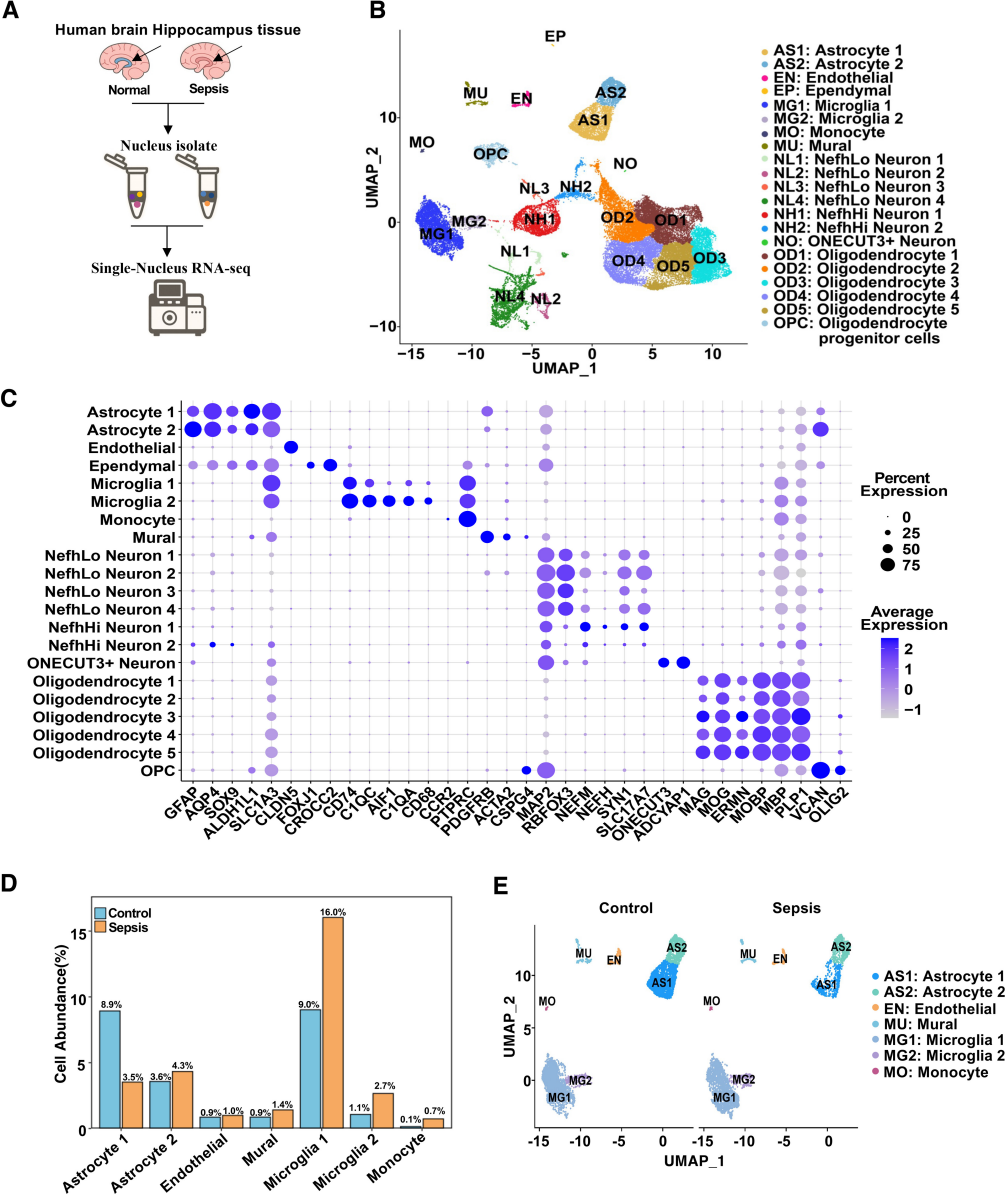
Single-nucleus RNA sequencing reveals cellular composition and transcriptional landscape of the human hippocampus in sepsis. **(A)** Schematic workflow of the snRNA-seq experiment. Human hippocampal tissues were collected postmortem, followed by nuclei isolation, library construction, sequencing, and computational analysis. **(B)** UMAP plot showing 21 transcriptionally distinct clusters identified across all samples, each annotated by cell type. **(C)** Dot plot showing the expression of canonical marker genes used for cell type annotation. Dot size indicates the percentage of cells expressing the gene in a given cluster, and color intensity represents average expression. **(D)** Bar plot showing the mean percent abundance of major cell types in control (blue) and sepsis (orange) samples. **(E)** UMAP plots colored by sample group, showing integrated NVU clusters across control and sepsis conditions.

To characterize the cellular heterogeneity of the human hippocampus under sepsis conditions, we performed single-nucleus RNA sequencing (snRNA-seq) on hippocampal tissues from control and sepsis patients (**Figure 1A**). After quality control and integration, UMAP dimensionality reduction revealed 21 distinct cell clusters (**Figure 1B**; **Supplementary Figure S1A**), corresponding to canonical cell types including excitatory neurons, inhibitory neurons, astrocytes, oligodendrocytes, microglia, endothelial cells, mural cells, among others. Cell types were identified by cluster-specific expression of established marker genes (**Figure 1C**). Quantitative analysis of cell-type proportions demonstrated substantial shifts in specific populations between the two groups. Sepsis samples displayed a notable increase in microglia and astrocytes, particularly a reduction in Astrocyte cluster 1, alongside a significant increase in clusters Microglia 1 and Microglia 2 (**Figure 1D**). These changes suggest glial activation and neuroinflammatory responses during sepsis (19, 24). To further delineate neurovascular-related changes, we visualized neurovascular-associated populations (astrocytes, microglia, endothelial cells, monocytes cells and mural cells) across disease states (**Figure 1E**). Astrocyte Cluster 1 in sepsis group appeared more tightly distributed in UMAP space compared to controls, potentially indicating reduced transcriptional heterogeneity or a convergence toward a uniform activation state under pathological conditions (19). Given these changes in the distribution and proportions of microglia and astrocyte clusters, we next examined subtype-specific transcriptional responses to sepsis.

### 2.2 Microglial transcriptional alterations in sepsis

**Figure 2:**
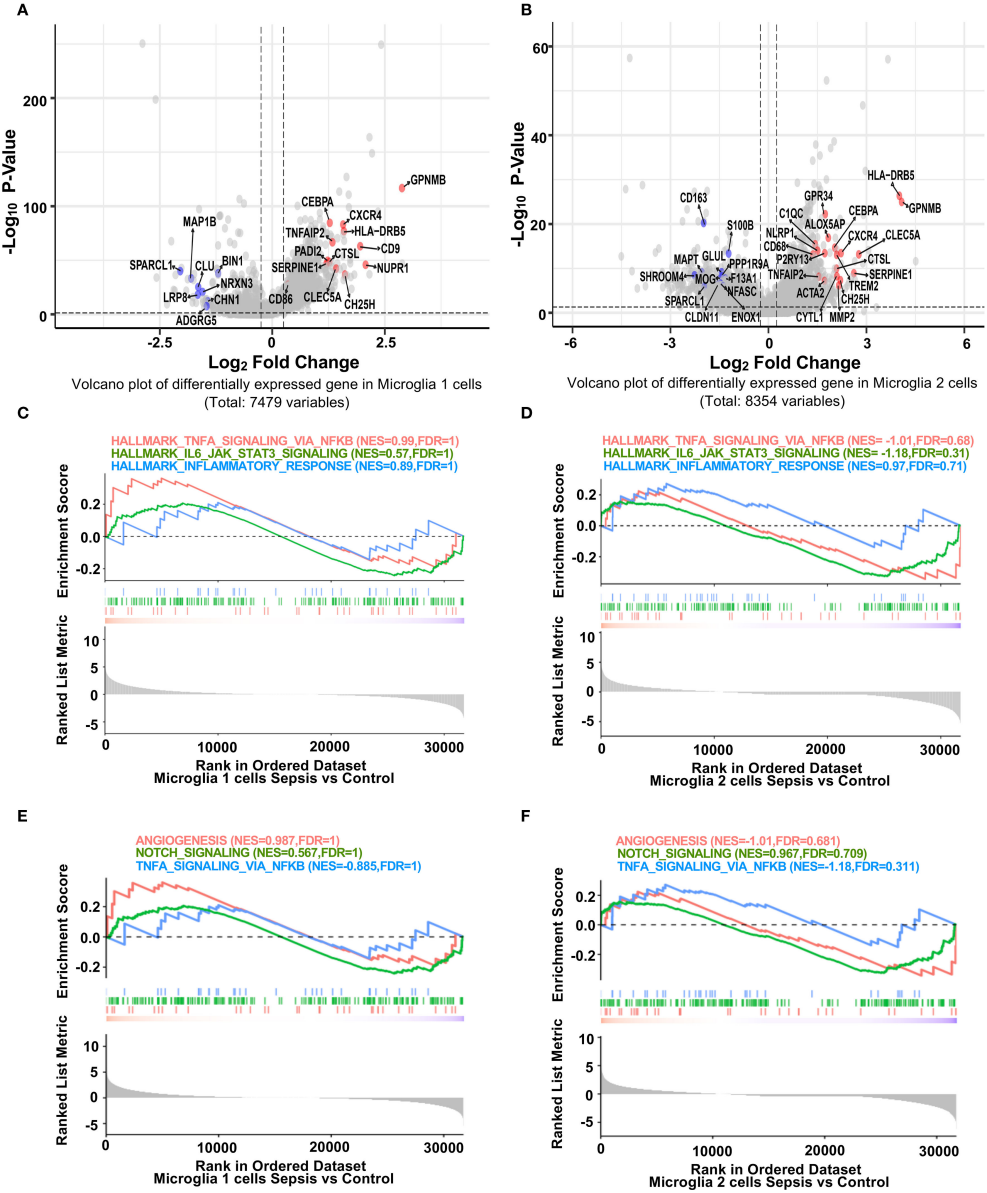
Distinct transcriptional responses of hippocampal microglia subpopulations to sepsis. **(A, B)** Volcano plots showing fold difference relative to significance for differentially expressed genes in Microglia 1 and Microglia 2 clusters between sepsis and control groups (Seurat Find Markers ()). Red-labeled genes are inflammation-related genes upregulated in sepsis; blue-labeled genes are blood-brain barrier (BBB)-associated genes downregulated in sepsis. Dotted lines indicate thresholds for significance (adjusted *p* < 0.05, |log_2_FC| > 0.25). **(C-F)** GSEA enrichment plots for selected hallmark pathways. Positive enrichment scores indicate pathway activation in transcripts with higher expression in sepsis. While inflammatory and immune-related pathways (e.g., TNF-α signaling via NF-κB, allograft rejection) show trends toward enrichment in sepsis microglia, they did not reach statistical significance in either microglial cluster.

To investigate microglial subtype-specific responses to sepsis, we conducted differential gene expression analysis between control and sepsis samples within Microglia 1 and Microglia 2. Volcano plots highlighted genes that were significantly upregulated (red; primarily inflammatory) or downregulated (blue; often blood-brain barrier–related) in sepsis (**Figures 2A–B**). Specifically, inflammatory genes such as *GPNMB* (25), *HLA-DRB5 (* 26), *CTSL*, *CXCR4*, *TNFAIP2*, and *CEBPA* were consistently upregulated in both microglial subsets. Conversely, BBB-associated genes including *SPARCL1*, *MAP1B*, *CALM1* (in Microglia 1) and *SPARCL1 (* 27), *CD163*, *MAPT*, *SHROOM4*, *GLUL* (in Microglia 2) were significantly downregulated. These expression signatures suggest that Microglia 1 represents a more pro-inflammatory subpopulation, while Microglia 2 maintains partial homeostatic features (28). Heatmaps of top 30 DEGs from each microglial subset further illustrated subtype-specific transcriptomic responses (**Supplementary Figure S2**, **S3**). GSEA analysis revealed that both Microglia 1 (**Figure 2C**) and Microglia 2 (**Figure 2D**) exhibited enrichment trends in hallmark inflammatory pathways, including TNF-α/NF-κB signaling (29), IL6–JAK–STAT3 signaling (30), and inflammatory response, although these did not reach statistical significance. Additionally, both subtypes (**Figures 2E, F**) showed non-significant enrichment trends in pathways related to blood-brain barrier function, such as angiogenesis, Notch signaling, and TNF-α/NF-κB signaling (31), though these did not reach statistical significance, indicating that while specific genes are highly dysregulated, transcriptional deregulation regarding these gene sets is heterogeneous.

### 2.3 Astrocytic inflammatory activation and BBB impairment

**Figure 3:**
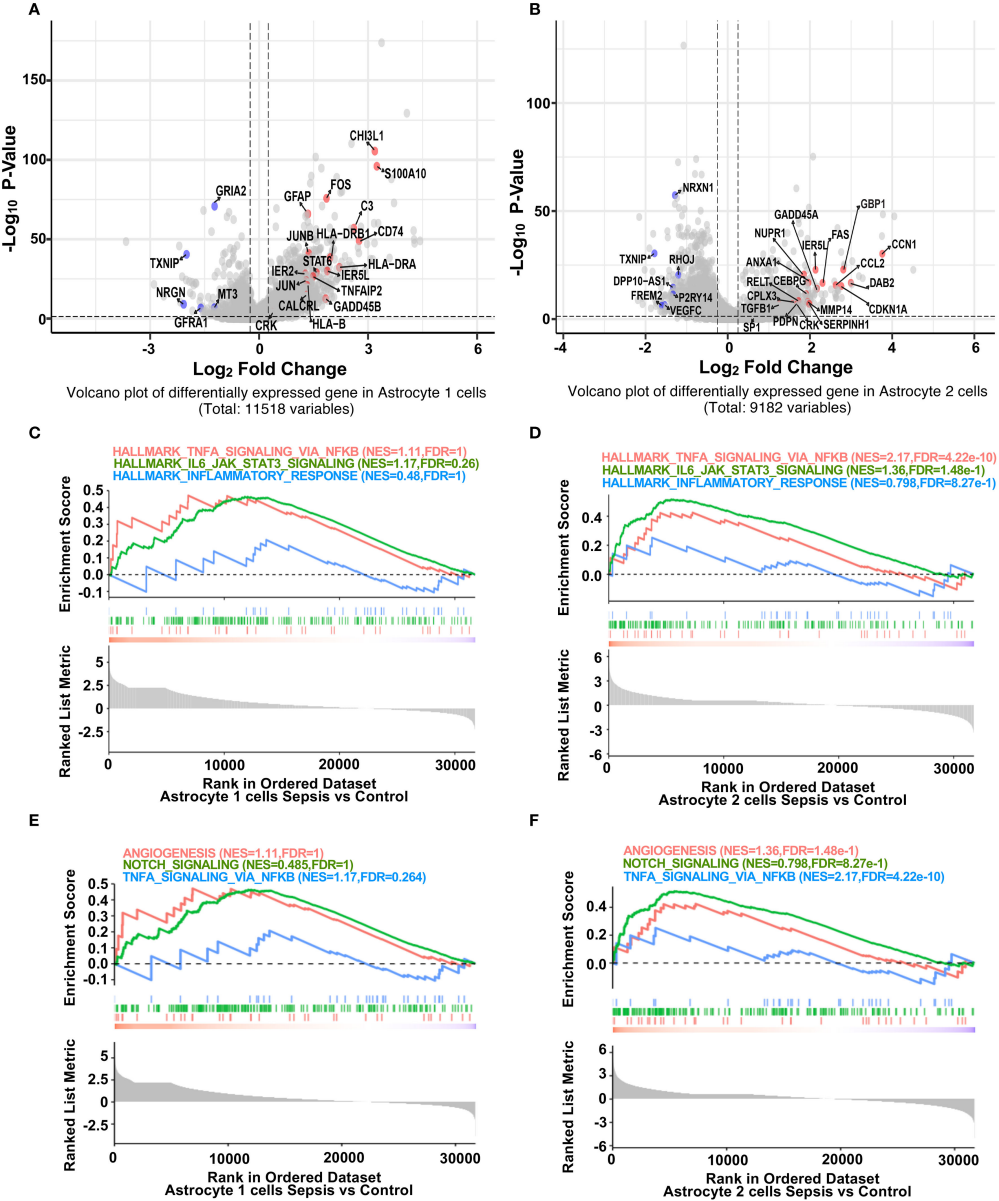
Sepsis induces inflammatory gene upregulation and impairs BBB-related pathways in astrocyte subpopulations. **(A)** Volcano plot showing differentially expressed genes in Astrocyte 1 between sepsis and control groups. Inflammation-related genes (red) were upregulated, while BBB-associated genes (blue) were downregulated after sepsis. **(B)** Volcano plot showing differentially expressed genes in Astrocyte 2 between sepsis and control groups. Similar to Astrocyte 1, inflammation-related genes were upregulated, and BBB-associated genes were downregulated. **(C)** GSEA plots for Astrocyte 1 showing enrichment of inflammatory pathways (Hallmark TNF-α Signaling via NF-κB, Hallmark IL6 Jak STAT3 Signaling, Hallmark_Inflammatory_Response) in the sepsis group. **(D)** GSEA plots for Astrocyte 2 showing enrichment of the same inflammatory pathways under sepsis conditions. **(E)** GSEA plots for Astrocyte 1 showing changes in BBB-related pathways (Angiogenesis, Notch Signaling, TNF-α Signaling via_ NF-κB) in the sepsis group. **(F)** GSEA plots for Astrocyte 2 illustrating the suppression of BBB-related pathways following sepsis.

We next explored transcriptional alterations in astrocyte subtypes. In Astrocyte 1, inflammation-associated genes (*GFAP*, *FOS*, *C3*, *CD74*) *(* 32, 33) were significantly upregulated, while BBB-related genes (*GRIA2*, *NRGN*, *TXNIP*) were downregulated in sepsis (**Figure 3A**) (19). Notably, *GRIA2* and *NRGN* are involved in astrocyte–neuron signaling and ion homeostasis, while *TXNIP* regulates redox balance and endothelial integrity. The downregulation of these genes suggests impaired astrocytic support for the neurovascular unit, potentially contributing to a barrier-disruptive phenotype and altered local hemodynamics. Similar but more pronounced changes were observed in Astrocyte 2, with upregulation of *CCL2 (* 34, 35), *FOS*, and *CEBPB*, and downregulation of *VEGFC* and *NRXN1* (**Figure 3B**). GSEA analysis revealed that both astrocyte subsets (Astrocyte 1 and 2) exhibited trends toward enrichment of hallmark inflammatory pathways, including TNF-α/NF-κB signaling, IL6–JAK–STAT3 (36), and inflammatory response (**Figures 3C, D**). In contrast, BBB-related pathways such as angiogenesis and Notch signaling (37) showed a consistent trend of downregulation in both subsets (**Figures 3E, F**), suggesting a shift toward pro-inflammatory and barrier-disruptive astrocyte states in sepsis. DEG heatmaps further confirmed subtype-specific transcriptional profiles (**Supplementary Figure S4**, **S5**). These data indicate that astrocytes undergo robust inflammatory activation and BBB-associated functional loss during sepsis.

### 2.4 Neurovascular unit-wide inflammatory activation

**Figure 4:**
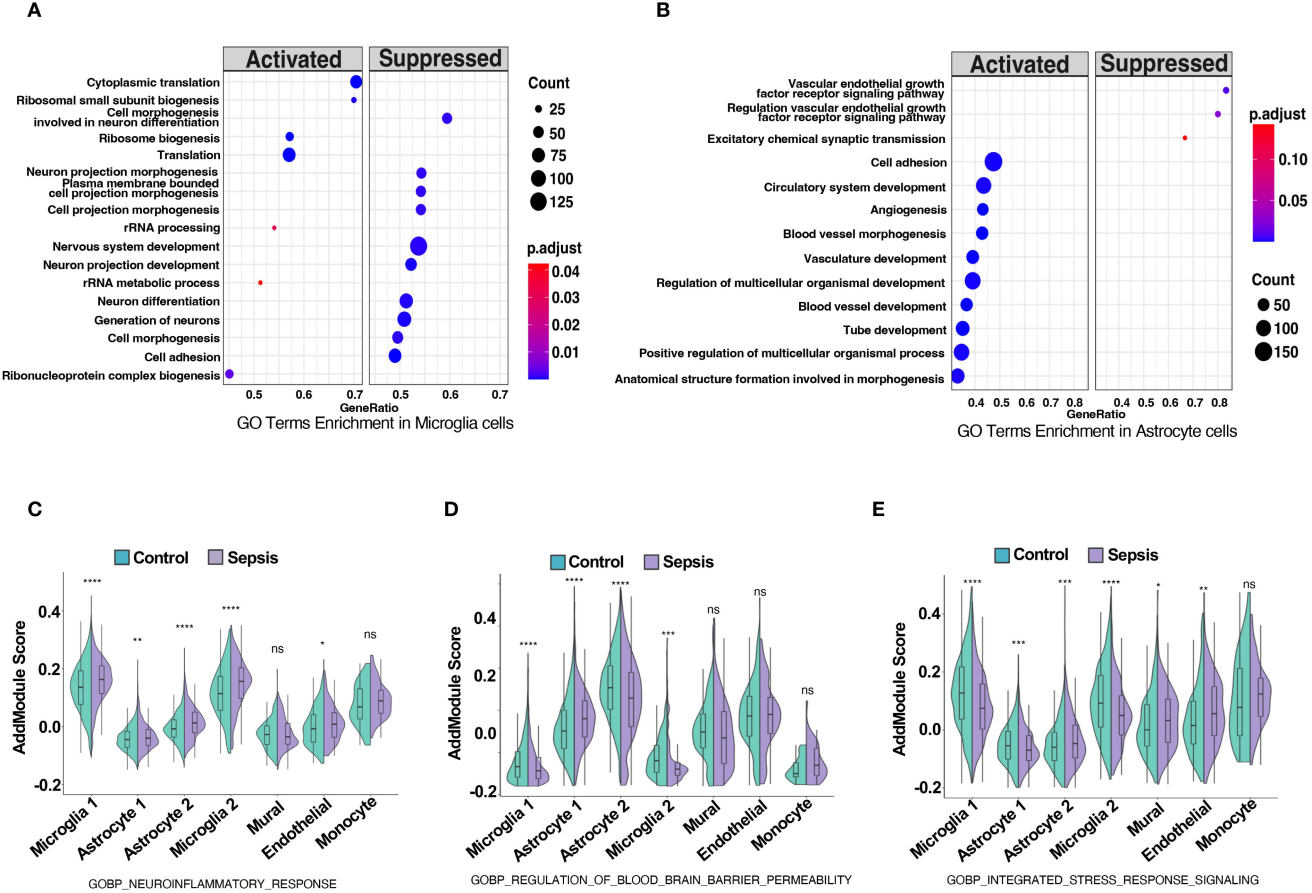
Sepsis induces neuroinflammation, blood-brain barrier dysregulation, and stress response activation in neurovascular-associated cell types. **(A,B)** GO term enrichment analysis based on differentially expressed genes in **(A)** microglia and **(B)** astrocytes, comparing sepsis versus control groups. Significantly enriched biological processes are shown, with dot color indicating adjusted p-value and size representing the number of genes per term. **(C–E)** Violin plots show module scores calculated using Seurat’s AddModuleScore for selected GO biological process (GOBP) gene sets in control (green) and sepsis (purple) samples across five neurovascular-related cell types: astrocytes, microglia, mural cells, endothelial cells, and monocytes. **(C)** Module scores for GOBP Neuroinflammatory Response are significantly elevated across all five cell types in sepsis. **(D)** Module scores for GOBP Regulation of Blood Brain Barrier Permeability are increased in astrocytes, microglia, and endothelial cells under sepsis, indicating potential BBB impairment. **(E)** Module scores for GOBP Integrated Stress Response Signaling are upregulated in astrocytes, mural cells, and endothelial cells, reflecting stress-related transcriptional adaptation. Statistical significance was evaluated using the Wilcoxon rank-sum test. ****p<0.0001, ***p<0.001, **p<0.01, *p<0.05. ns = not significant.

GO terms enrichment analysis revealed neurovascular unit-wide immune dysregulation and blood–brain barrier (BBB)-related pathway alterations. In microglia, upregulated pathways such as cytoplasmic translation, ribosome biogenesis and rRNA metabolic processes may indicate increased metabolic or biosynthetic activity. However, whether these changes reflect immune-related translational activation or a broader shift in baseline rRNA dynamics remains unclear and warrants further investigation (38). (**Figure 4A**). Concurrently, key pathways involved in neuron differentiation, projection morphogenesis, and cell adhesion were downregulated, suggesting impaired microglial support for neuronal structure and function within the neurovascular context. In astrocytes, enriched GO terms highlighted activation of vascular remodeling and BBB-associated processes, including angiogenesis, blood vessel morphogenesis, and multicellular organismal development (39) (**Figure 4B**). In contrast, downregulated pathways involved the vascular endothelial growth factor receptor (VEGFR) signaling and excitatory synaptic transmission, indicating disrupted neurovascular signaling and BBB integrity. These findings indicate substantial transcriptional shifts in astrocytes, particularly involving proinflammatory activation and reduced expression of genes associated with neurovascular support. Such changes may influence the integrity and functionality of the neurovascular interface, which will be further explored in downstream intercellular communication analyses. Module scoring with curated GO gene sets showed significantly elevated scores for neuroinflammatory response (GOBP Neuroinflammatory Response) in microglia, astrocytes, and endothelial cells (**Figure 4C**). Additionally, BBB permeability regulation (GOBP Regulation of Blood Brain Barrier Permeability) and integrated stress response signaling were significantly elevated across neurovascular cell types (**Figures 4D, E**). Additionally, gene set enrichment analysis (GSEA) in endothelial and mural cells showed upregulation of inflammation-related pathways and downregulation of vascular homeostasis in sepsis (40) (**Supplementary Figure S1B, C**), establishing a foundation for cell-type-specific transcriptional analyses. DEG heatmaps for endothelial and mural cells corroborated these functional shifts (**Supplementary Figure S6**, **S7**), indicating coordinated yet cell-type-specific adaptations to systemic sepsis.

### 2.5 Sepsis induces divergent polarization states in microglial subpopulations

**Figure 5:**
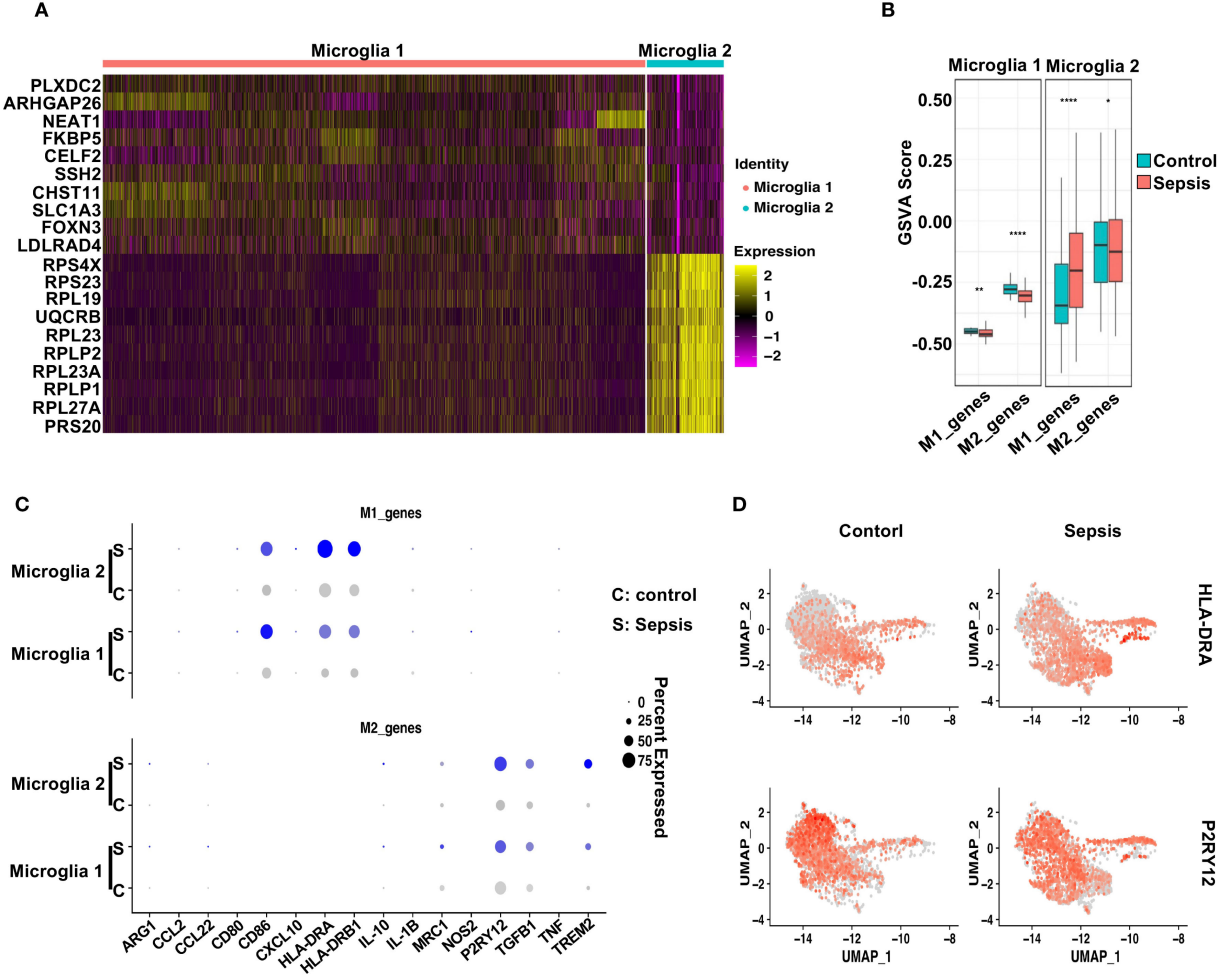
Sepsis induces distinct polarization states in microglial subtypes. **(A)** Heatmap displaying the top 10 differentially upregulated genes in Microglia 1 and Microglia 2. **(B)** GSVA scores comparing M1- and M2-associated gene signatures across Control and Sepsis conditions in Microglia 1 and Microglia 2. Microglia 1 exhibited reduced M2 scores in Sepsis, while Microglia 2 showed increased M1 scores. **(C)** Dot plot showing the expression levels and percentage of cells expressing individual M1 markers and M2 markers in Microglia 1 and Microglia 2 under Control and Sepsis conditions. **(D)** Feature plots highlighting the spatial expression of HLA-DRA (M1 marker) and P2RY12 (M2 marker) across microglial clusters, illustrating subtype-specific transcriptional polarization.

To dissect the immunological reprogramming of microglia in sepsis, we performed a detailed comparative analysis between Microglia 1 and Microglia 2 subclusters. Heatmap visualization of differentially expressed genes (DEGs) revealed distinct transcriptional signatures between the two populations (**Figure 5A**). Among the top genes enriched in Microglia 1 were *PLXDC2*, *ARHGAP26*, *NEAT1* (41), *FKBP5* (42), and *CELF2* (43), which have been associated with stress response, transcriptional regulation, and homeostatic maintenance. In contrast, Microglia 2 exhibited high expression of *RPS4X*, *RPS23*, *RPL19*, *RPL23*, and *UQCRB*, suggesting enhanced ribosomal biogenesis and mitochondrial activity, potentially reflective of increased translational demand during inflammatory activation.

To further characterize their polarization status, we conducted gene set variation analysis (GSVA) using curated M1- and M2-associated gene sets (44). M1/M2 polarization was inferred by applying GSVA to predefined gene signatures (see Methods), rather than dynamic modeling systems. Thus, the observed trends reflect relative gene set activity rather than definitive functional polarization states. In Microglia 1, sepsis induced a modest decrease in M1-related gene scores and a marked reduction in M2-associated signatures compared to controls (**Figure 5B**), indicating a shift away from a homeostatic M2-like phenotype. In contrast, Microglia 2 exhibited a robust increase in M1 gene scores alongside a slight reduction in M2 scores in sepsis samples, consistent with a pro-inflammatory M1-like polarization. Dot plot analysis highlighted the expression patterns of individual M1 and M2 marker genes across both subtypes and conditions (**Figure 5C**). Notably, *HLA-DRA*, *IL1B*, and *CD86* were prominently elevated in Microglia 2 from sepsis patients, while *P2RY12* and *TREM2* were reduced, further supporting a pro-inflammatory shift (38, 45–47). Feature plots of *HLA-DRA* and *P2RY12* underscored their subtype- and condition-specific expression patterns (**Figure 5D**). Together, these findings suggest that sepsis drives a bifurcation of microglial states: Microglia 2 adopts a classical M1-like pro-inflammatory profile, while Microglia 1 displays features of stress adaptation but loses M2-associated homeostatic identity, reflecting a dual mode of immune activation and glial dysfunction (48–50).

### 2.6 Sepsis promotes subtype-specific astrocyte polarization in coordination with microglial activation

**Figure 6:**
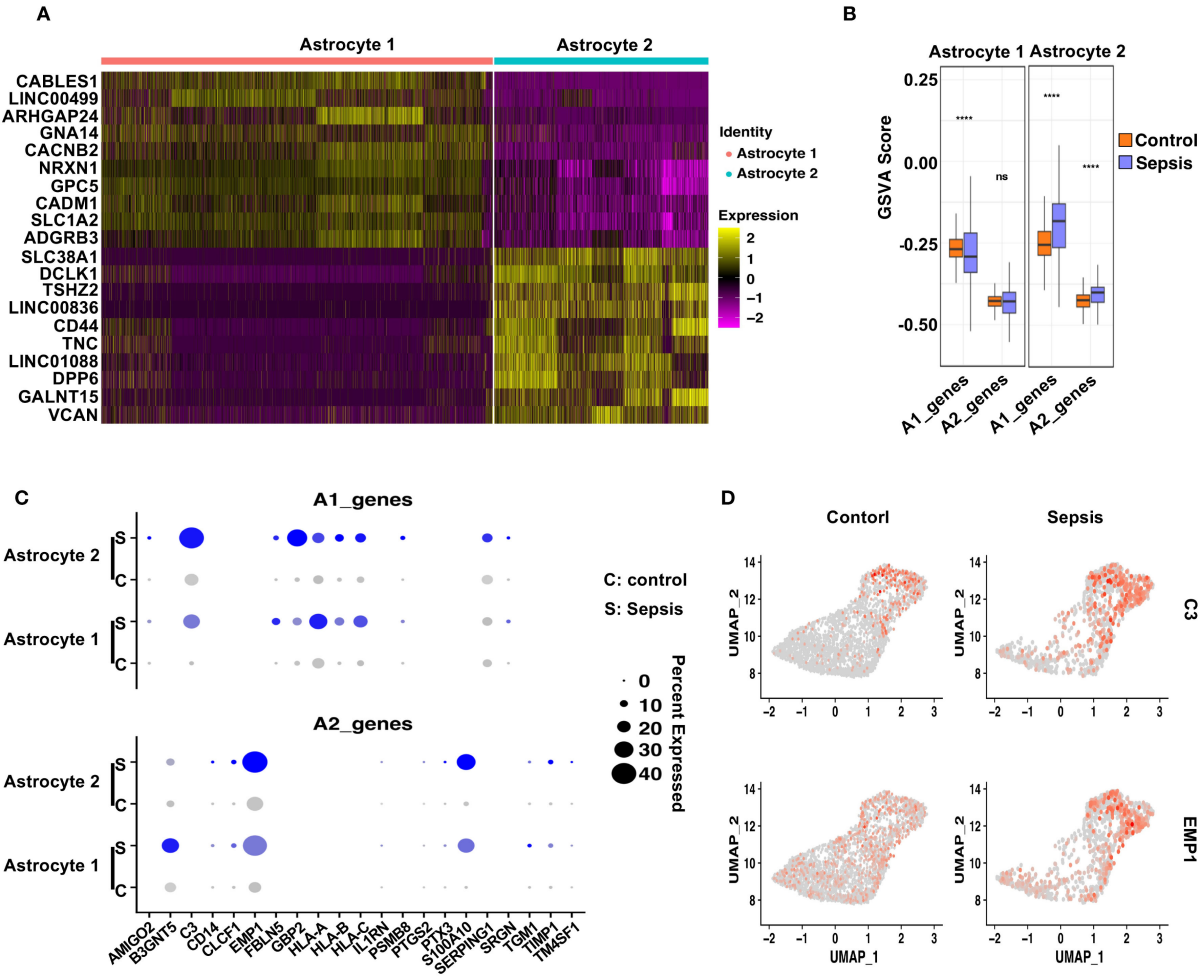
Distinct astrocyte subpopulations exhibit divergent polarization patterns following sepsis. **(A)** Heatmap showing the top 10 differentially upregulated genes in Astrocyte 1 and Astrocyte 2. **(B)** GSVA analysis of A1- and A2-associated gene sets in Astrocyte 1 and Astrocyte 2 under Control and Sepsis conditions. Astrocyte 1 showed a decrease in A1 scores in Sepsis, whereas Astrocyte 2 displayed significant upregulation of both A1 and A2 gene signatures. **(C)** Dot plot showing the expression of individual A1 and A2 genes in both astrocyte subtypes, across conditions. **(D)** Feature plots displaying representative A1 (C3) and A2 (EMP1) markers, demonstrating their distinct spatial expression in astrocyte subclusters.

To evaluate astrocytic responses to sepsis, we analyzed transcriptional profiles of Astrocyte 1 and Astrocyte 2 subpopulations. Heatmap analysis revealed clear transcriptomic divergence between the two clusters (**Figure 6A**). Astrocyte 1 preferentially expressed genes such as *CABLES1*, *LINC00499*, *ARHGAP24*, *GNA14*, and *CACNB2*, many of which are involved in cytoskeletal remodeling and calcium signaling. In contrast, Astrocyte 2 was enriched for genes including *SLC38A1 (* 51), *DCLK1 (* 52), *TSHZ2*, and *CD44*, suggesting enhanced roles in synaptic modulation, reactive transformation, and cell–cell interaction. To interrogate their polarization dynamics, we performed GSVA using curated gene sets representing neurotoxic (A1) and neuroprotective (A2) reactive astrocyte signatures. In sepsis samples, Astrocyte 1 showed a significant decrease in A1-related scores and no significant change in A2 scores compared to controls, indicating a dampened reactive profile (**Figure 6B**). Conversely, Astrocyte 2 exhibited a robust increase in both A1 and A2 gene set scores upon sepsis, suggestive of a hybrid reactive state encompassing both pro-inflammatory and potentially reparative programs (19, 53, 54). Dot plot visualization further confirmed these trends at the gene level (**Figure 6C**), with *C3*, *SERPING1*, and *HLA-A/B/C* being notably upregulated in Astrocyte 2 under sepsis conditions, while *EMP1*, *CLCF1*, and *PTGS2*—representing A2 features—were similarly elevated. Feature plots for *C3* and *EMP1* highlighted their distinct expression patterns across astrocyte subtypes and disease states (**Figure 6D**).

These findings, when integrated with the microglial data, suggest a coordinated glial polarization pattern during sepsis. Specifically, the emergence of a pro-inflammatory Astrocyte 2 population parallels the activation of M1-like Microglia 2, while the relatively quiescent Astrocyte 1 and Microglia 1 subsets may represent stress-adapted but functionally compromised states. This subtype-specific astrocyte–microglia interaction may contribute to blood-brain barrier (BBB) disruption and sustained neuroinflammation in the septic brain, offering potential cellular targets for therapeutic intervention.

### 2.7 Remodeling of microglia–neurovascular communication networks

**Figure 7:**
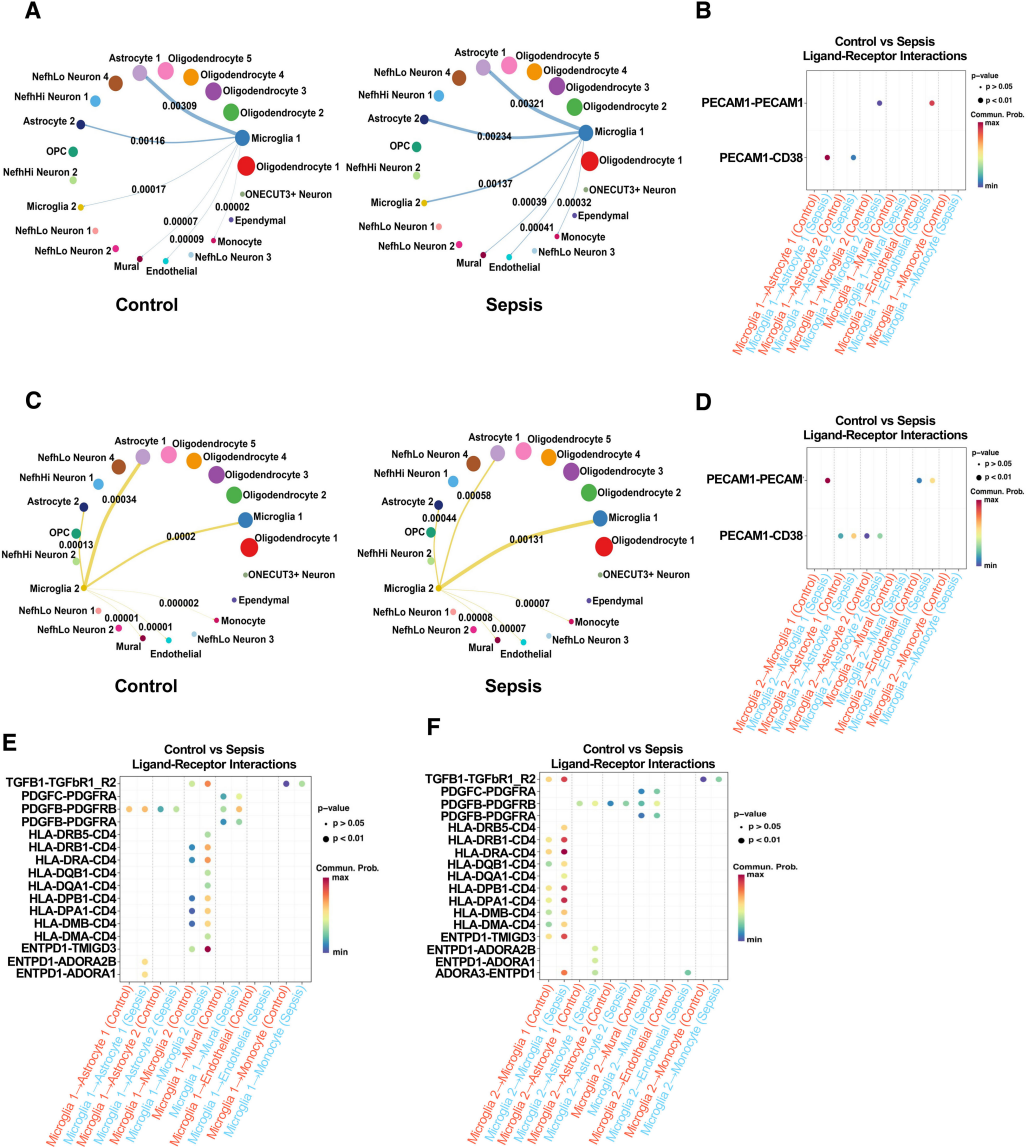
Sepsis remodels microglial intercellular communication with neurovascular-associated cells via inflammatory and BBB-associated signaling pathways. **(A)** CellChat analysis of Microglia 1 outgoing signaling to neurovascular-related cells (Microglia 2, Astrocyte 1, Astrocyte 2, endothelial, mural, and monocytes) in control (left) and sepsis (right) groups. Sepsis increases the number and strength of outgoing connections from Microglia 1. **(B)** Comparison of inflammation-related ligand–receptor pair interactions from Microglia 1 to neurovascular cells under control (blue) and sepsis (red) conditions. Sepsis induces stronger pro-inflammatory interactions, including TGF-β, PDGF, and Antigen presentation and immune response and Adenosinergic signaling pathways. **(C)** CellChat analysis of Microglia 2 outgoing communication to neurovascular-related cells in control (left) and sepsis (right) groups, showing enhanced intercellular communication in sepsis. **(D)** Sepsis-induced changes in inflammation-related ligand–receptor pair interactions from Microglia 2 to neurovascular-associated cells. **(E)** Sepsis increases Microglia 1 communication through BBB-associated ligand–receptor pairs directed toward endothelial and mural cells. **(F)** Similar but less prominent changes in BBB-related ligand–receptor interactions are observed in Microglia 2 under sepsis conditions.

To investigate microglial interactions with neurovascular-associated cells, we performed CellChat analysis. Sepsis enhanced the outgoing communication strength (i.e., ligand enrichment) from Microglia 1 toward astrocytes, endothelial cells, mural cells, and monocytes. In contrast, Microglia 2 showed a significant increase in interactions with Microglia 1, while interactions with Astrocyte 1 were weakened. Interactions with mural cells, endothelial cells, and monocytes were also enhanced, although not significantly. (**Figures 7A, C**). Analysis of inflammatory ligand–receptor interactions revealed increased signaling through TGF-β, PDGF, and Antigen presentation and immune response and Adenosinergic signaling pathways (**Figures 7B, D**), while sepsis-induced enhanced interactions between Microglia 1 and Astrocytes 1 and 2 via PECAM1-CD38, and between Microglia 1 and Endothelial cells and Microglia 2 via PECAM1-PECAM1, suggesting increased neuroinflammation (55). Similarly, Microglia 2 showed enhanced PECAM1-CD38 interactions with Astrocytes 1 and 2 and PECAM1-PECAM1 interactions with Endothelial cells and Microglia 1. These changes likely compromise blood-brain barrier integrity, increasing permeability and promoting the infiltration of inflammatory cells (**Figures 7E, F**). Microglia 1 exhibited a stronger increase in both inflammatory and BBB-disruptive communications compared to Microglia 2.

### 2.8 Astrocyte–neurovascular communication dynamics

**Figure 8:**
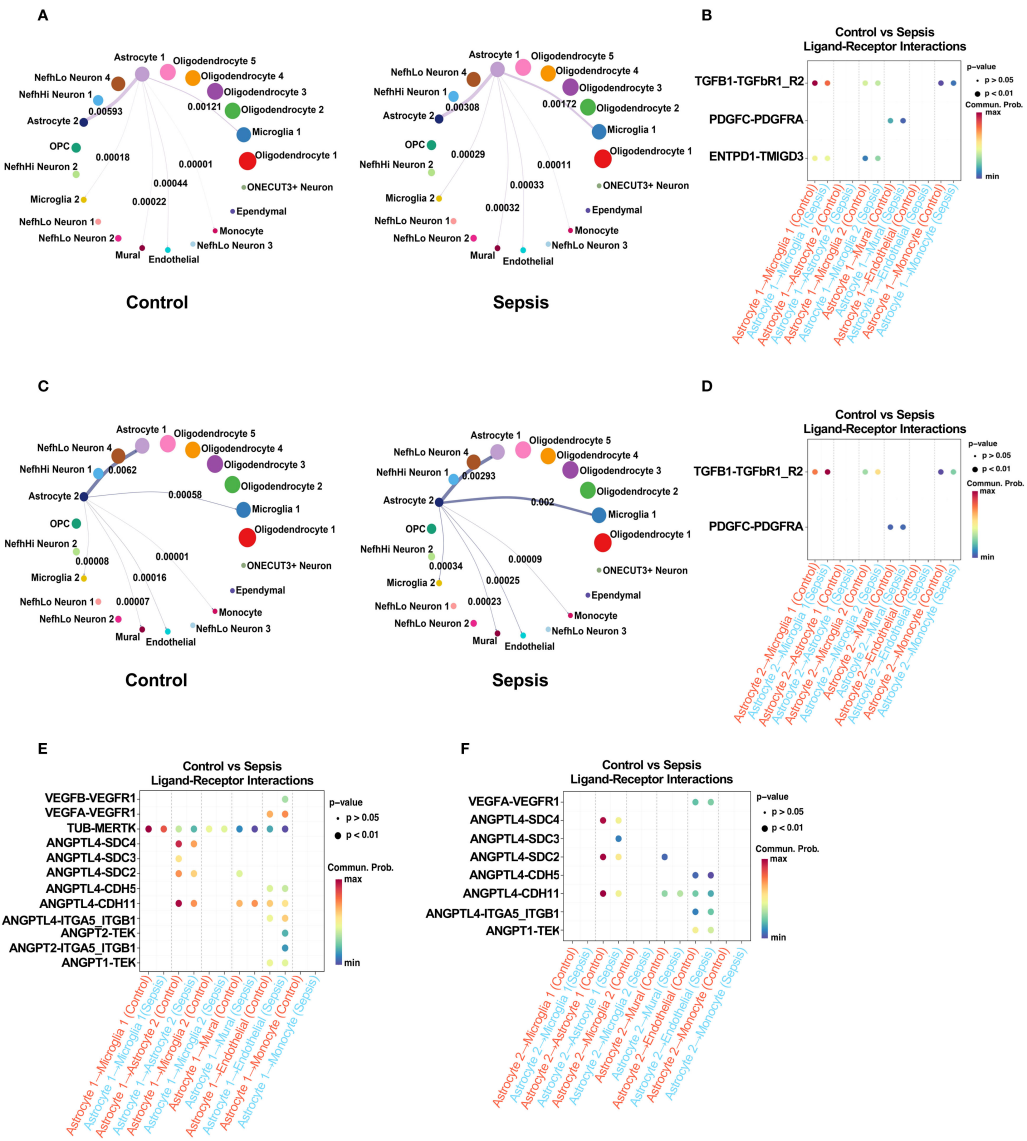
Sepsis alters astrocyte intercellular communication with neurovascular-associated cells via inflammatory and BBB-related signaling pathways. **(A)** CellChat analysis of outgoing communication from Astrocyte 1 to neurovascular-associated cells (Astrocyte 2, Microglia 1, Microglia 2, endothelial cells, mural cells, and monocytes) in control (left) and sepsis (right) groups. Sepsis increases the strength and breadth of outgoing signals from Astrocyte 1. **(B)** Comparison of inflammation-related ligand–receptor interactions derived from Astrocyte 1 toward neurovascular-associated cells between control and sepsis groups. Sepsis enhances pro-inflammatory signaling, notably through TNF, IL6, and BMP pathways. **(C)** CellChat analysis of outgoing communication from Astrocyte 2 to neurovascular-associated cells in control (left) and sepsis (right) groups, showing amplified signaling in the sepsis condition. **(D)** Comparison of inflammation-related ligand–receptor interactions derived from Astrocyte 2 toward neurovascular-associated cells. Sepsis leads to increased inflammatory communication involving TGF-β and PDGF pathways. **(E)** Comparison of BBB-associated ligand–receptor interactions from Astrocyte 1 to neurovascular-associated cells in control and sepsis groups. Sepsis upregulates interactions such as ANGPT2–TEK, VEGFA–FLT1, and CXCL12–CXCR4. **(F)** BBB-associated ligand–receptor interactions from Astrocyte 2 to neurovascular-associated cells show a sepsis-induced increase, although to a lesser extent compared with Astrocyte 1.

CellChat analysis of astrocytes revealed that both Astrocyte 1 and Astrocyte 2 displayed markedly increased outgoing signaling toward microglia, mural, and endothelial cells after sepsis (**Figures 8A, C**). Inflammatory ligand–receptor interactions, particularly involving TGF-β (56), PDGF pathways, were upregulated in sepsis samples (**Figures 8B, D**). Additionally, BBB-modulatory interactions such as ANGPT2–TEK and VEGFA–FLT1 (57) were significantly enhanced in both astrocyte subtypes (**Figures 8E, F**). These results suggest that astrocytes, like microglia, actively remodel their communication networks under sepsis, promoting inflammatory signaling and BBB disruption.

**Figure 9:**
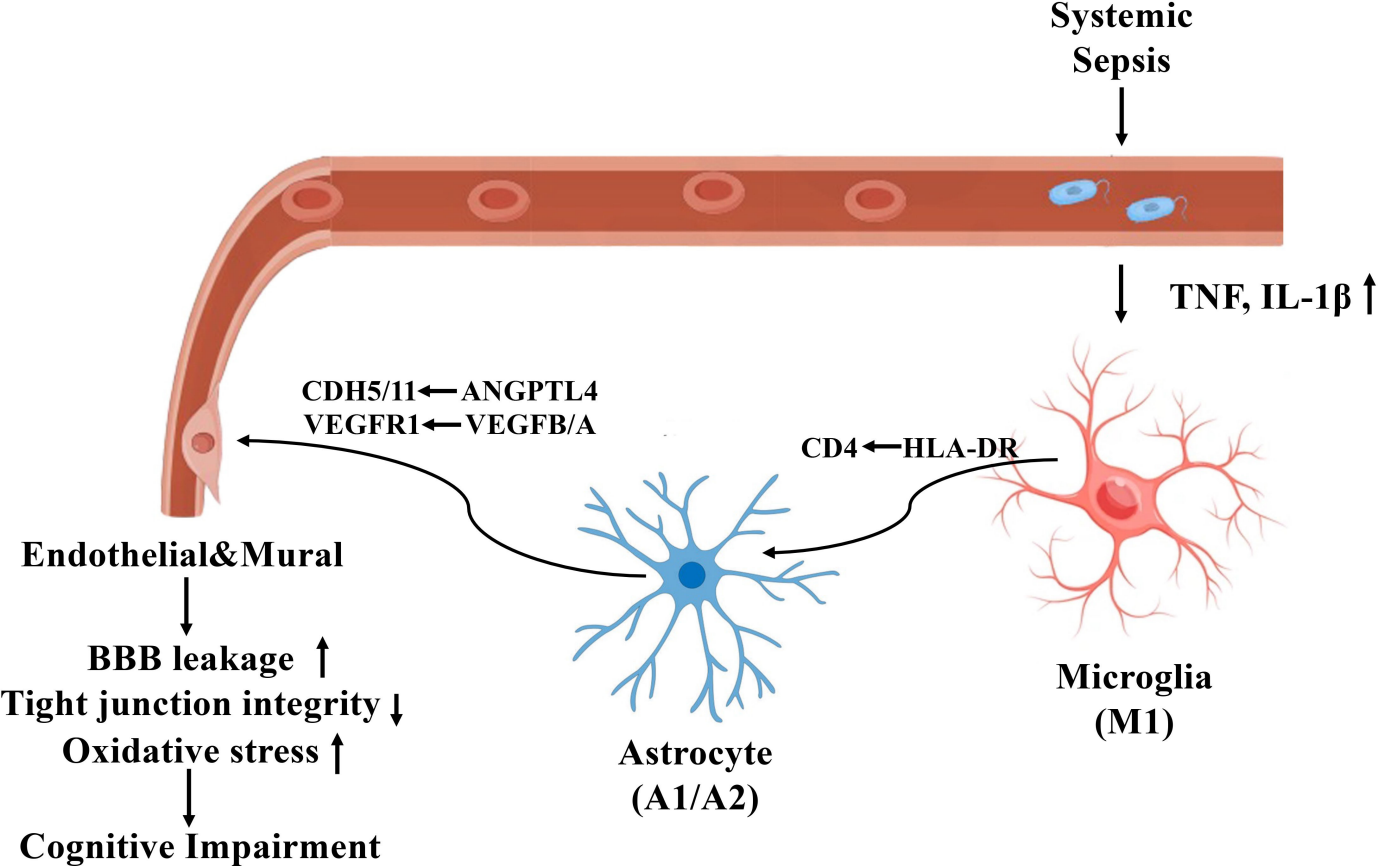
Mechanistic illustration of sepsis-induced neurovascular dysfunction and cognitive impairment in the human hippocampus.

**Figure 9** illustrates the proposed mechanism by which sepsis induces neurovascular dysfunction in the human hippocampus, highlighting glial activation, blood-brain barrier disruption, and maladaptive glial-vascular interactions that collectively contribute to cognitive impairment.

## 3 Discussion

Sepsis-associated encephalopathy (SAE) is a frequent but underrecognized complication of systemic infection, characterized by acute and chronic cognitive impairments. Although neuroinflammation and blood-brain barrier (BBB) dysfunction are implicated, the precise cellular and molecular mechanisms underlying SAE remain incompletely understood (10, 58). In this study, we utilized single-nucleus RNA sequencing (snRNA-seq) to systematically characterize hippocampal cell-type-specific transcriptional alterations in *postmortem* tissues from sepsis and control patients. Our results provide a high-resolution map of the neurovascular unit (NVU) remodeling during sepsis, revealing profound inflammatory activation, BBB-associated gene dysregulation, and intercellular communication perturbations among microglia and astrocyte subpopulations (59). Microglia are central to CNS innate immunity, and their activation states critically influence neuroinflammation and BBB integrity. We identified two transcriptionally distinct microglia subtypes (Microglia 1 and Microglia 2), both of which expanded in proportion and displayed distinct transcriptional programs following sepsis. In sepsis, Microglia 1 exhibited strong upregulation of pro-inflammatory genes (e.g., *GPNMB*, *CXCR4*, *HLA-DRB5*) and downregulation of BBB-supportive genes (e.g., *SPARCL1*, *MAP1B*), indicating a highly reactive phenotype. In contrast, Microglia 2 maintained partial homeostatic features but still demonstrated significant transcriptional remodeling, including upregulation of *TNFAIP2* and downregulation of *MAPT* and *CD163*, consistent with partial transition toward a disease-associated phenotype.

This dichotomy was further supported by GSVA-based M1/M2 polarization analysis, revealing loss of M2 signatures in Microglia 1 and acquisition of M1 features in Microglia 2. These observations align with previous reports demonstrating microglial heterogeneity in neurodegenerative and neuroinflammatory contexts, including Alzheimer’s disease and traumatic brain injury (60–62). Notably, prior immunohistochemistry and imaging studies have shown that these glial subtypes tend to localize near blood vessels or at sites of neuroinflammation (59, 63, 64). We acknowledge the limitation of lacking direct spatial information in our dataset and have cited key studies supporting these localization patterns. Furthermore, we recognize that incorporating spatial transcriptomics or multiplexed immunostaining would be an valuable future direction to more definitively map these subpopulations in human sepsis brain tissue. Similarly, astrocyte subtypes exhibited divergent polarization patterns, with Astrocyte 2 co-expressing A1 and A2 signatures, while Astrocyte 1 displayed attenuated expression of both, suggestive of a context-dependent reactive state. Differential expression analysis further revealed that genes upregulated in Microglia 2 (e.g., *RPS23*, *UQCRB*) and Astrocyte 2 (e.g., *SLC38A1*, *DCLK1*) were enriched for ribosomal and metabolic functions, whereas Microglia 1 and Astrocyte 1 expressed stress- or immune-regulatory genes such as *PLXDC2*, *FKBP5*, and *ARHGAP24*. This molecular divergence raises the possibility that Microglia 2 and Astrocyte 2 may be preferentially localized to more inflamed or perivascular niches, where intense immune activation and BBB breakdown occur, whereas Microglia 1 and Astrocyte 1 may reside in relatively protected or adaptive compartments. Although spatial validation is warranted, these findings highlight a coordinated, spatially compartmentalized glial response to systemic inflammation, contributing to the persistence of hippocampal dysfunction in sepsis.

In this study, we assessed the polarization states of microglia and astrocytes by applying gene set variation analysis (GSVA) based on established M1-like (pro-inflammatory) and M2-like (anti-inflammatory or reparative) gene signatures. These analyses allowed us to infer the relative enrichment of classical versus alternative activation programs within glial subclusters. However, we acknowledge that this binary M1/M2 classification oversimplifies the complex spectrum of glial activation observed *in vivo*, particularly under pathophysiological conditions such as sepsis.

The polarization states of glial cells are highly plastic, context-dependent, and influenced by local microenvironmental cues, including cytokines, metabolic stress, and vascular integrity. Furthermore, transcriptomic signatures may not fully reflect the functional state of glia without validation at the protein level or spatial context. For example, certain anti-inflammatory gene programs may be transiently upregulated as compensatory responses, rather than indicating stable alternative activation. Therefore, our results should be interpreted as indicative of relative activation biases rather than definitive functional assignments. Future studies incorporating protein-based validation or spatial transcriptomics will be essential to confirm and refine our understanding of glial polarization dynamics in the septic brain.

Astrocytes, critical regulators of the BBB and CNS homeostasis, also exhibited robust transcriptional reprogramming (19). In Astrocyte 1 and Astrocyte 2 subtypes, we observed upregulation of inflammatory mediators (*C3*, *GFAP*, *CD74*, *FOS*) and downregulation of genes supporting BBB integrity (*NRGN*, *GRIA2*, *TXNIP*, *VEGFC*). In Astrocyte 2, GSEA confirmed significant enrichment of inflammatory pathways (TNF-α/NF-κB, IL6–JAK–STAT3), along with repression of angiogenesis and Notch signaling, highlighting a dual program of neuroinflammation and BBB breakdown. Interestingly, our astrocyte polarization analysis identified a shift toward A1-reactive states—particularly in Astrocyte 2—consistent with neurotoxic astrocyte activation described in models of sepsis, ischemia, and neurodegeneration. In addition, Astrocyte 2 increased both in overall abundance and in particular as a percentage of astrocytes.

A major strength of our analysis lies in the comprehensive evaluation of the NVU, including microglia, astrocytes, endothelial cells, and mural cells. GO and module score analyses revealed that sepsis induced widespread enrichment of neuroinflammatory responses and stress-related pathways across all neurovascular cell types. In parallel, genes and pathways associated with extracellular matrix organization, cell adhesion, and BBB permeability regulation were consistently disrupted across cell types. These findings provide transcriptional evidence supporting structural and functional compromise of the BBB during SAE. Our results extend prior histological and bulk transcriptomic studies showing endothelial cell dysfunction and pericyte detachment during sepsis, offering new insights into cell-type-specific gene expression programs underlying these processes.

Using CellChat analysis, we found that microglia and astrocytes increased their outgoing signaling to endothelial and mural cells during sepsis, particularly through inflammatory ligand-receptor pairs such as TGF-β, PDGF, and adenosinergic signaling (65, 66). Notably, PECAM1-mediated interactions were markedly enhanced between microglia, astrocytes, and endothelial cells—an axis previously implicated in leukocyte transmigration and vascular permeability in systemic inflammation (67). The differential communication profiles of Microglia 1 versus Microglia 2, as well as of the two astrocyte subtypes, further support the idea of cell-state-dependent contributions to BBB disruption (38, 68). We conclude that intrinsic transcriptional reprogramming during sepsis reshapes the inflammatory microenvironment through paracrine signaling interactions, ultimately impacting the integrity of the NVU.

Our study highlights that SAE involves complex, cell-type- and subtype-specific transcriptional changes that coordinate to produce a state of neurovascular dysfunction and inflammation. These findings underscore the importance of targeting not only individual cell types but also the intercellular communication networks that drive neuroinflammatory cascades. Therapeutic strategies aimed at modulating glial activation states (69) (e.g., restoring M2 or A2 phenotypes), blocking inflammatory signaling (e.g., TGF-β, PECAM1), or reinforcing BBB structural integrity may hold promise for mitigating CNS complications in sepsis.

Limitations of our study include the small sample size and reliance on *postmortem* tissue, which may not fully capture dynamic disease trajectories. Nonetheless, the depth of single-nucleus resolution provides valuable insights that complement and extend animal model findings. Further integration with proteomic, spatial transcriptomic, and functional assays will be essential to validate and translate these molecular signatures into therapeutic targets (70).

## 4 Materials and methods

### 4.1 Human hippocampal tissue collection

*Postmortem* human hippocampal brain tissues were obtained from the NIH NeuroBioBank and the Medical University of South Carolina (MUSC) Brain Bank. Samples were collected from six individuals: three diagnosed with sepsis and three age-matched control subjects. The control group included two females (ages 88 and 82) and one male (age 58), while the sepsis group included two females (ages 83 and 78) and one male (age 61). None of the individuals had a documented history of neurodegenerative disease. The use of *postmortem* human tissue was approved by the respective Institutional Review Boards (IRBs) of MUSC.

### 4.2 Single-nucleus RNA sequencing and initial data processing

Frozen tissue samples were shipped to Novogene (San Jose, CA), where nuclei were isolated by homogenizing the tissue, filtering the homogenate to remove debris, and pelleting the nuclei by centrifugation. Single-cell 3′ RNA libraries were prepared using the 10x Genomics Chromium Single Cell 3′ v3.1 platform. Libraries were sequenced on an Illumina NovaSeq X Plus system with a target depth of approximately 50,000 read pairs per nucleus. Initial data processing was performed by Novogene, including alignment to the GRCh38 human reference genome. Raw sequencing data were further processed using Cell Ranger (71) (10x Genomics) with default parameters, including unique molecular identifier (UMI) collapsing, barcode filtering, and generation of gene expression matrices.

### 4.3 Filtering, normalization, integration, and clustering

Subsequently, batch correction and downstream analyses were performed using the Seurat v5.0.1 (72). Cells with fewer than 200 detected genes, more than 5,000 genes, or over 10% mitochondrial gene content were filtered out to remove low-quality or dying cells. Genes expressed in fewer than 3 cells were also excluded. Hippocampal snRNA-seq samples were normalized using SCTransform (73) (vst.flavor = “v2”, return.only.var.genes = FALSE) with regression of sequencing depth, mitochondrial transcript percentage, and complexity and retaining all genes. Shared genes were identified (SelectIntegrationFeatures) and integration features were centered and scaled (PrepSCTIntegration). Highly variable genes were identified and used for dimensionality reduction via principal component analysis (PCA) and integration anchors were identified by FindIntegrationAnchors (reduction = “rpca”, dims = 1:50, k.anchor = 20. Samples were integrated using IntegrateData (normalization.method = “SCT”, dims = 1:50, k.weight = 100).

PCA was run again on the integrated dataset using RunPCA (npcs = 50), the SNN graph was constructed using FindNeighbors, and UMAP was constructed using PC. Clusters were identified by FindClusters (algorithm = 1, n.iter = 100) at a range of resolutions; resolution of 0.7 was selected based on cluster stability in clustree plots. The resulting clusters represent transcriptionally distinct subpopulations showing enrichment in established cell-specific markers. The data were then normalized using the “LogNormalize” method with a scaling factor of 10,000.

Quality control metrics, including the number of detected genes per cell, UMI counts, and mitochondrial gene percentages, were visualized using Dimplot in each sample to confirm the removal of low-quality cells and correction for technical covariates such as depth and complexity.

### 4.4 Marker gene identification and cell type annotation

To identify marker genes for each cluster, differential gene expression analysis was performed using the Wilcoxon rank-sum test implemented in the FindAllMarkers function of the Seurat package. Genes with an adjusted p-value (q-value) < 0.05 and log_2_ fold change > 0.25 were considered significantly differentially expressed. Cluster-specific marker genes were then compared against known canonical markers to assign cell-type identities to each cluster.

### 4.5 Differential expression and gene set enrichment analysis

Differentially expressed genes (DEGs) between sepsis and control groups were identified within specific cell types using Seurat’s FindMarkers() function (min.pct = 0.1 and logfc.threshold = 0), meaning that all genes expressed in at least 10% of cells were tested regardless of fold change. Genes with adjusted p < 0.05 and |log2 fold change| > 1 were considered significant. Gene Set Enrichment Analysis (GSEA) (74) was conducted using the fgsea (75) R package against MSigDB (76) Hallmark gene sets and curated gene ontology (GO) terms related to inflammation and blood–brain barrier (BBB) function. Gene set variation analysis (GSVA) (77) was used to calculate M1/M2 or A1/A2 polarization scores using pre-defined gene lists.

In this study, we employed both Gene Set Enrichment Analysis (GSEA) and Gene Set Variation Analysis (GSVA) to gain complementary insights into pathway-level changes between conditions. GSEA is a rank-based method that identifies whether predefined gene sets show statistically significant differences between two biological states at the population level and is particularly well-suited for identifying pathways enriched among differentially expressed genes. On the other hand, GSVA is a non-parametric, unsupervised method that calculates enrichment scores for each gene set in each individual sample or cell cluster, enabling sample-wise or cluster-wise pathway activity profiling. By combining these two approaches, we aimed to capture both group-level directional changes (via GSEA) and cell-type– or cluster–specific variation (via GSVA). This dual strategy enhances robustness and interpretability by cross-validating pathway trends from different analytical angles.

### 4.6 Ligand–receptor interaction analysis

Cell–cell communication networks were inferred using the CellChat (v2.1.2) package (78). Normalized expression data from Seurat objects were input into CellChat to estimate incoming and outgoing communication probabilities among neurovascular-related cell types. Communication probability matrices were calculated for both control and sepsis groups using the computeCommunProb and aggregateNet functions. Significant ligand–receptor pairs (p < 0.05) were visualized by heatmaps and network circle plots. Comparisons of interaction strength between groups were performed using the compareInteractions() function.

### 4.7 Gene ontology and pathway scoring

Module scores for biological processes such as neuroinflammatory response, regulation of BBB permeability, and integrated stress response were calculated using Seurat’s AddModuleScore function based on curated GO biological process (GOBP) gene sets. Statistical significance between control and sepsis groups was assessed using two-tailed Wilcoxon tests.

### 4.8 Data visualization

All data visualizations including UMAP plots, violin plots, heatmaps, volcano plots, dot plots, and network diagrams were generated using ggplot2, ComplexHeatmap (79), and CellChat’s built-in functions in R (v4.4.1).

## Data Availability

The original contributions presented in the study are publicly available. The raw and processed scRNA-seq data associated with our manuscript have been deposited in the NCBI Gene Expression Omnibus (GEO) database. This data can be found here: [https://www.ncbi.nlm.nih.gov/geo/query/acc.cgi?acc=GSE307512].
