# Supplementary material for: Single-nucleus transcriptomics reveals sepsis-related neurovascular dysfunction in the human hippocampus
Source: Front Immunol. 2025 Sep 15;16:1648278. doi: 10.3389/fimmu.2025.1648278 (PMC12477015; doi:10.3389/fimmu.2025.1648278)
Supplement: Supplementary file 1 [file DataSheet1.pdf]

Single-nucleus transcriptomics reveals sepsis-related neurovascular dysfunction in the human hippocampus

Liu Liu[1], Pengfei Li[1], Brent Wilkerson[2], Yan Wu[3], Meng Liu[3], Wei Jiang[4], Eric D. Hamlett[1], Steven L. Carroll[1], and Hongkuan Fan[1]*

*** Correspondence:**
Hongkuan Fan
fanhong@musc.edu

Figure S1

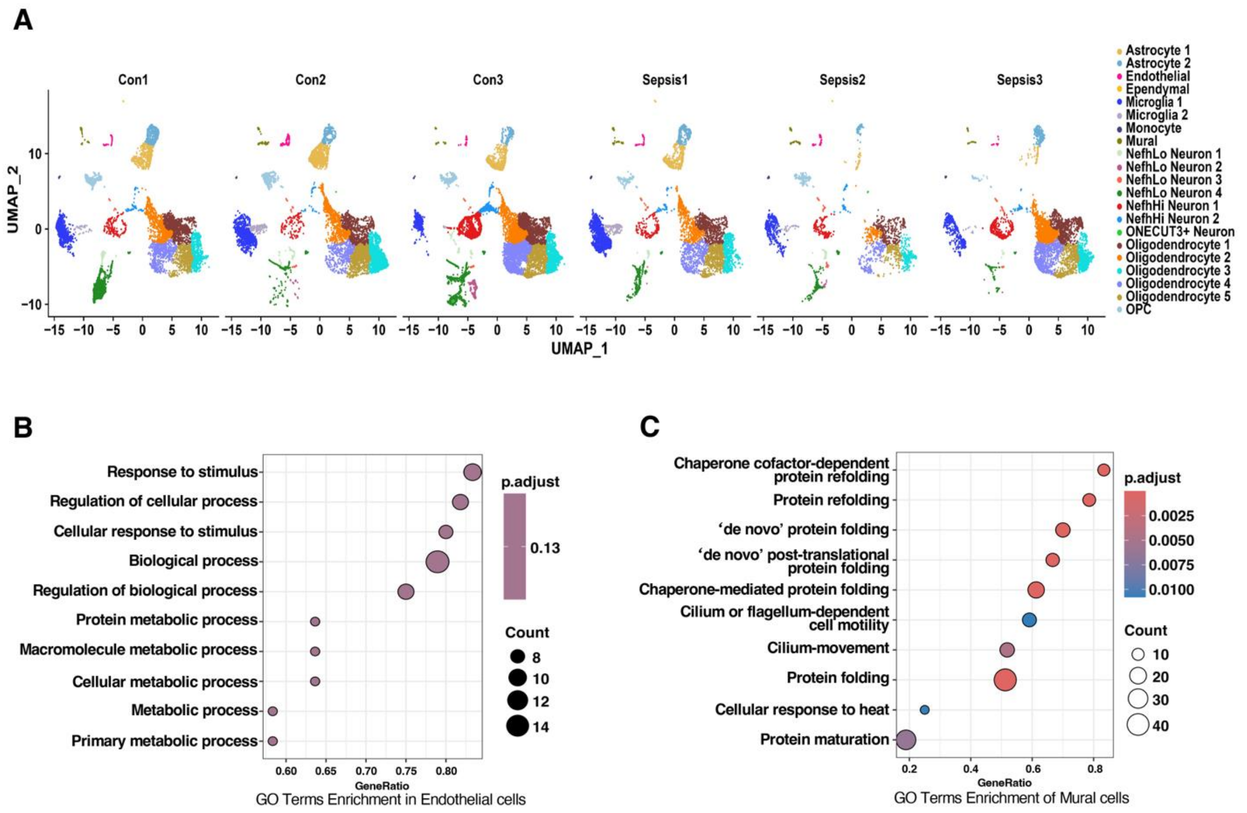

Figure S1. (A) UMAP plot showing 21 transcriptionally distinct cell clusters identified across six hippocampal samples, including three control and three sepsis cases. **(B, C)** GO term enrichment analysis of differentially expressed genes in **(B)** Endothelial and **(C)** Mural cells between sepsis and control groups. Significantly enriched biological processes are visualized, with dot color representing the adjusted *p*-value and dot size indicating the number of genes associated with each term.

Figure S2

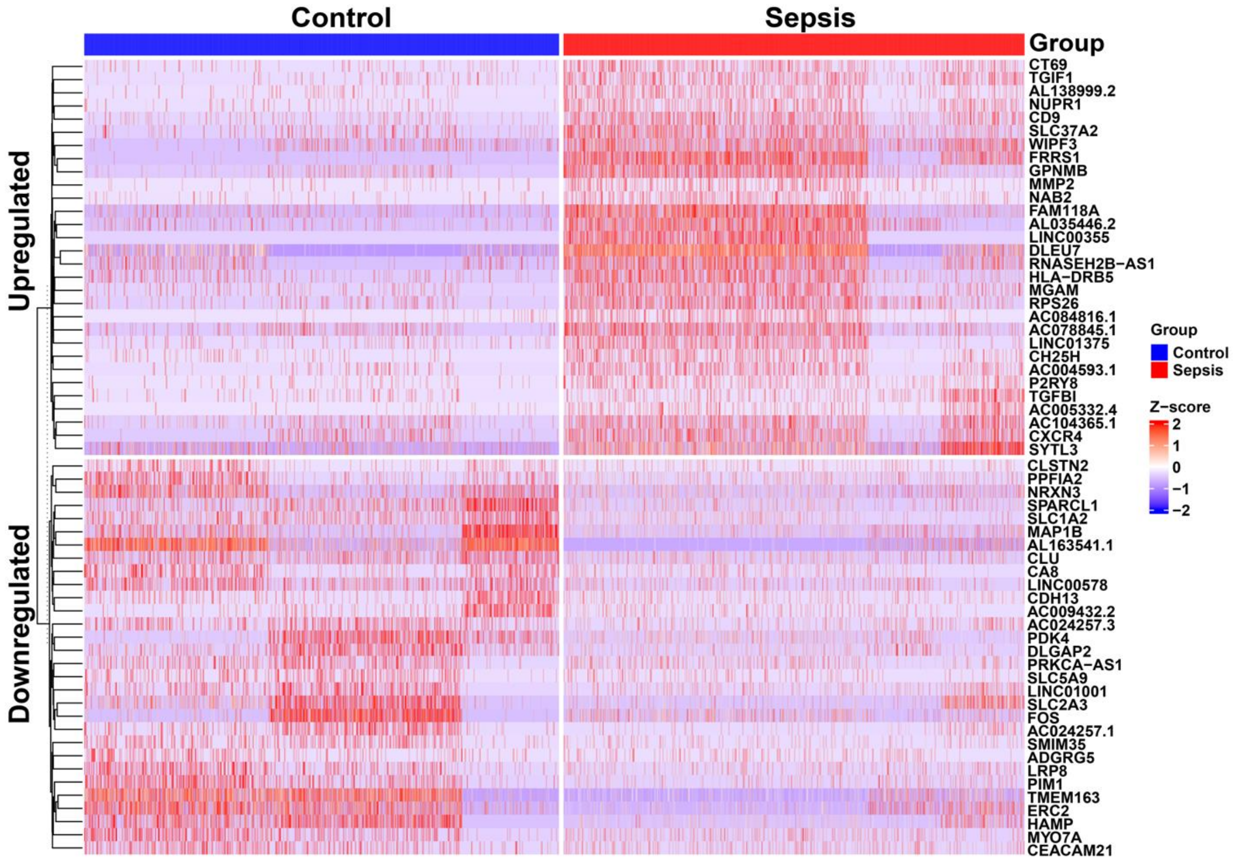

TOP 30 differentially expressed gene in Microglia 1 cells

Figure S2. Heatmap showing the top 30 differentially expressed genes (DEGs) in Microglia 1 cells when comparing sepsis and control groups. Genes were ranked by average log fold change, with upregulated and downregulated genes included.

Figure S3

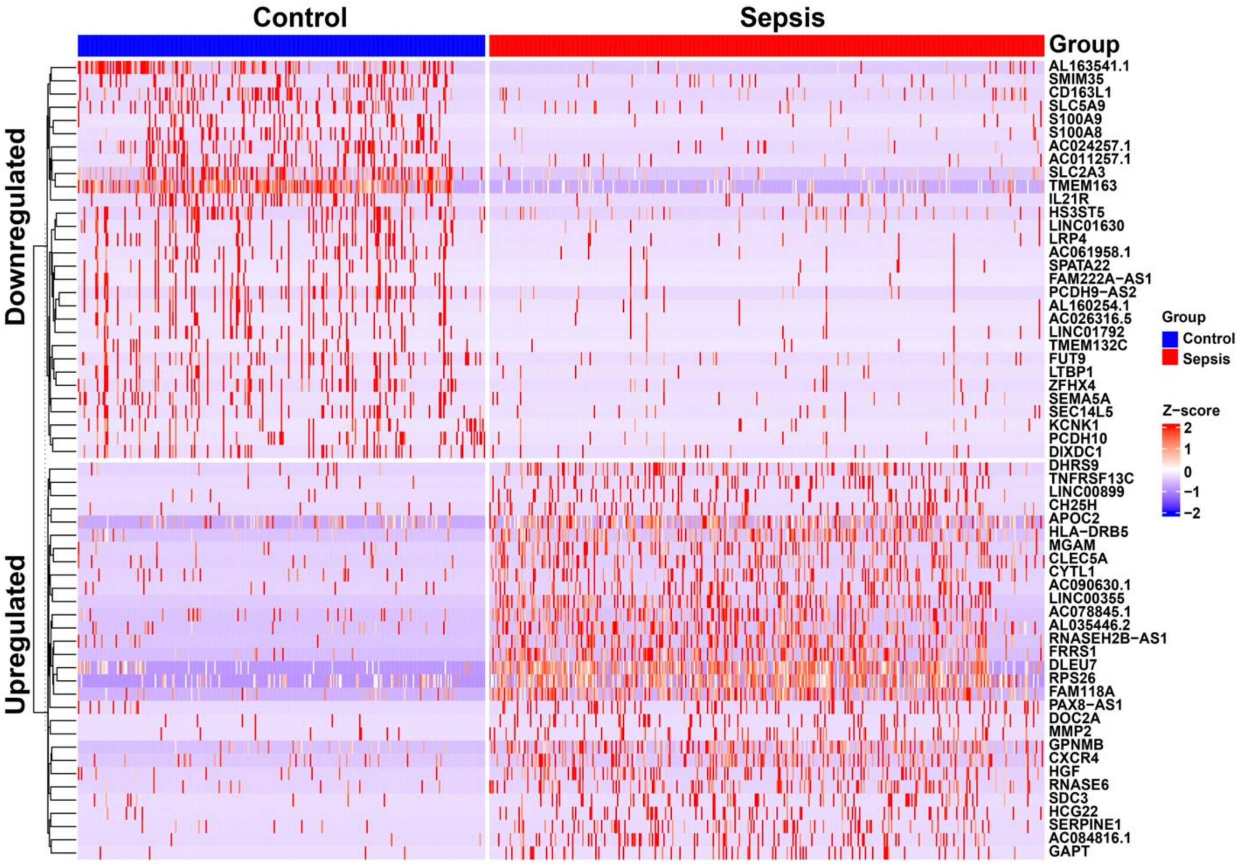

TOP 30 differentially expressed gene in Microglia 2 cells

Figure S3. Heatmap showing the top 30 differentially expressed genes (DEGs) in Microglia 2 cells when comparing sepsis and control groups. Genes were ranked by average log fold change, with upregulated and downregulated genes included.

Figure S4

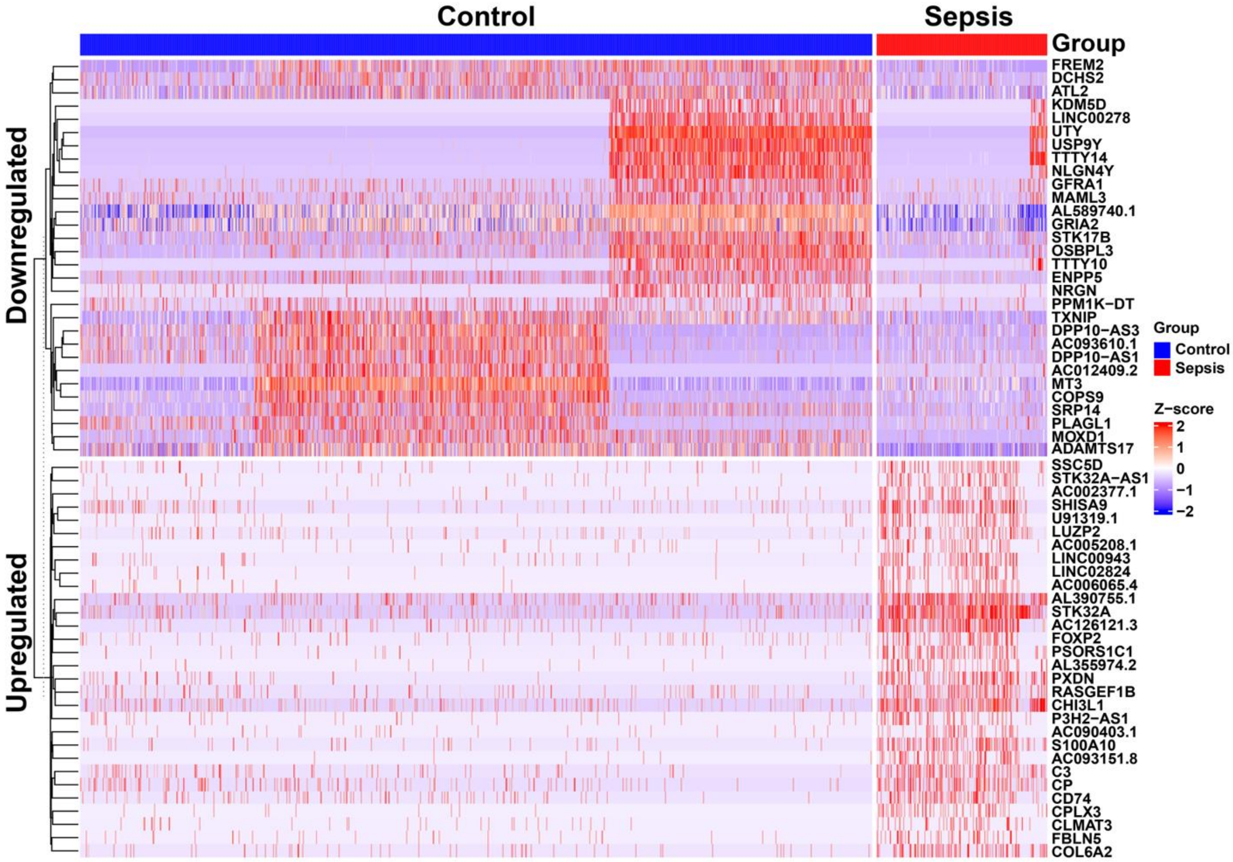

TOP 30 differentially expressed gene in Astrocyte 1 cells

Figure S4. Heatmap showing the top 30 differentially expressed genes (DEGs) in Astrocyte 1 cells when comparing sepsis and control groups. Genes were ranked by average log fold change, with upregulated and downregulated genes included.

Figure S5

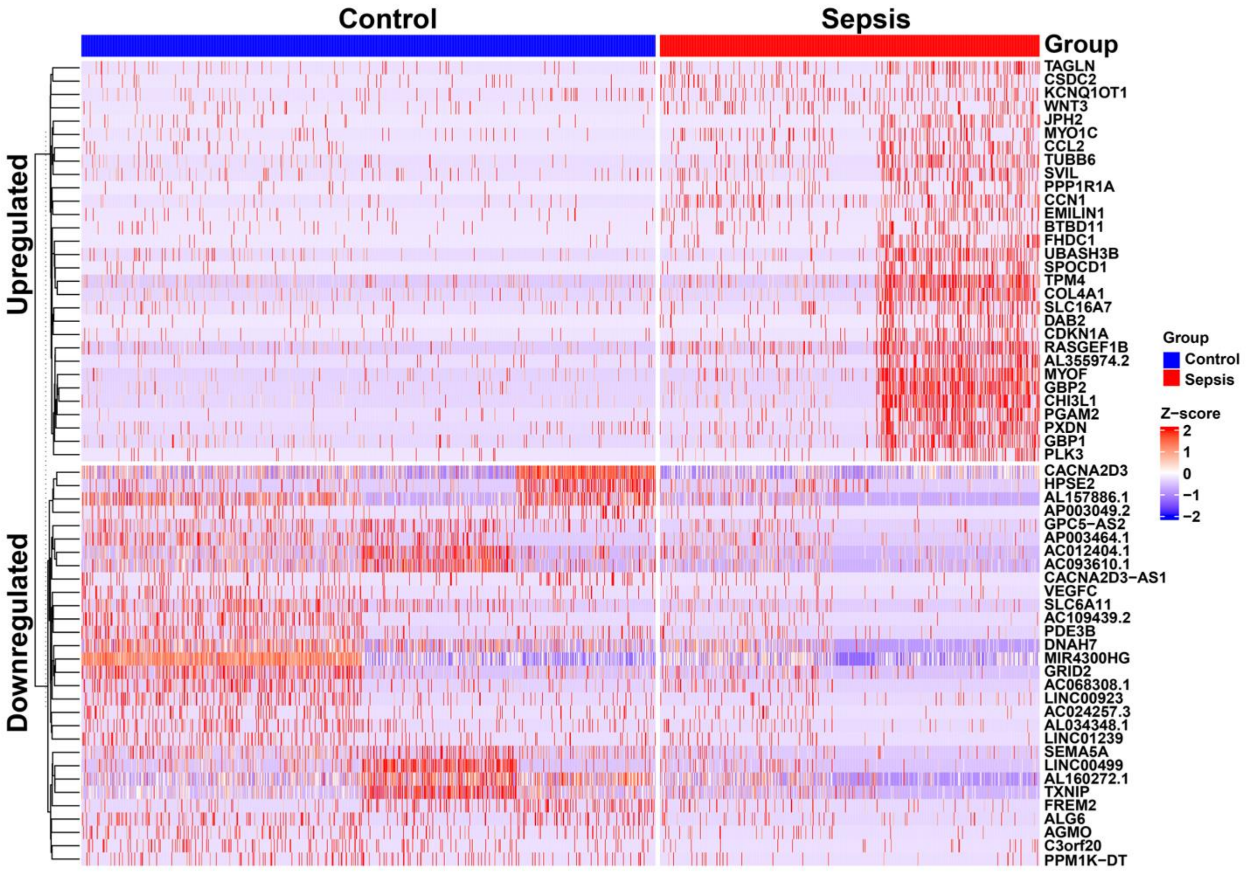

TOP 30 differentially expressed gene in Astrocyte 2 cells

Figure S5. Heatmap showing the top 30 differentially expressed genes (DEGs) in Astrocyte 2 cells when comparing sepsis and control groups. Genes were ranked by average log fold change, with upregulated and downregulated genes included.

Figure S6

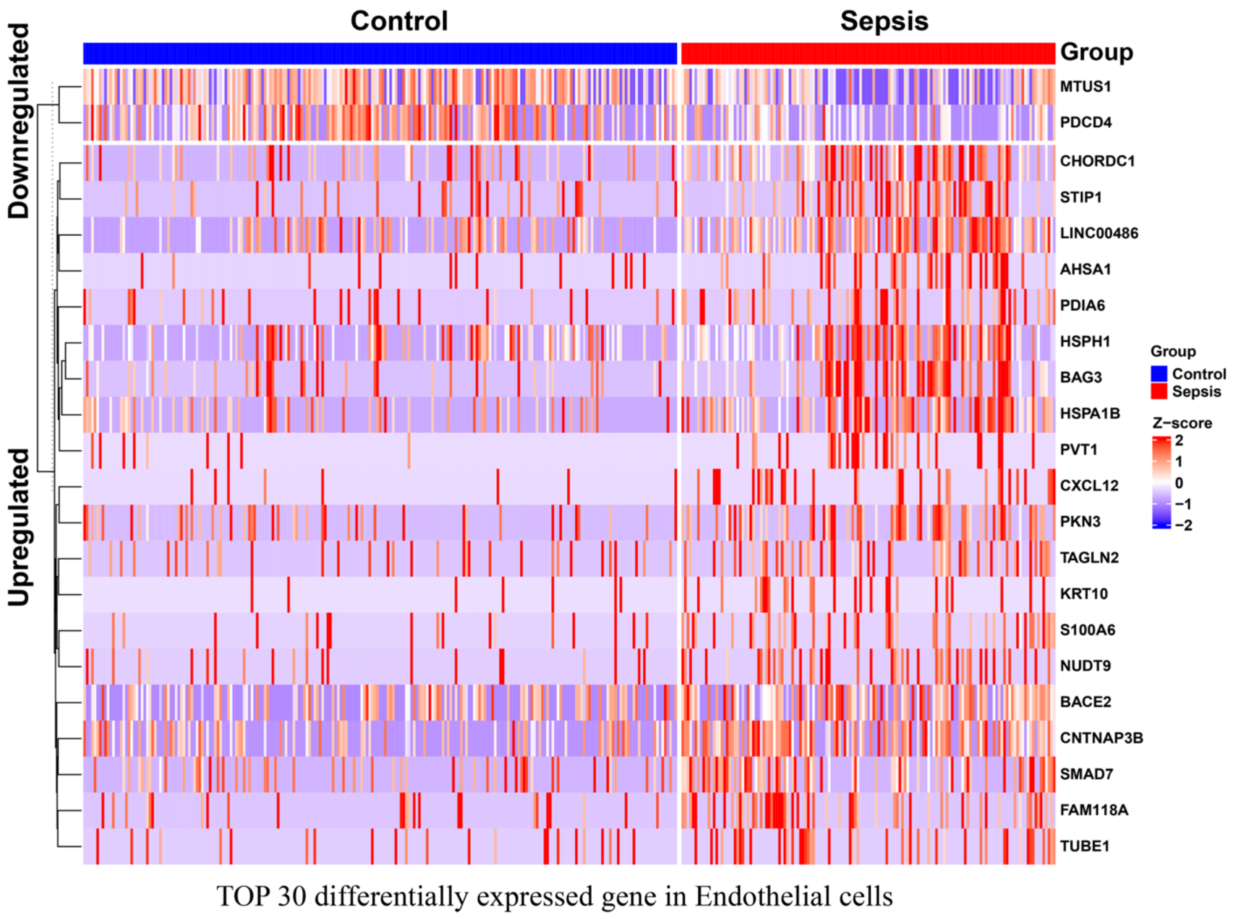

Figure S6. Heatmap showing the top 30 differentially expressed genes (DEGs) in Endothelial cells when comparing sepsis and control groups. Genes were ranked by average log fold change, with upregulated and downregulated genes included.

Figure S7

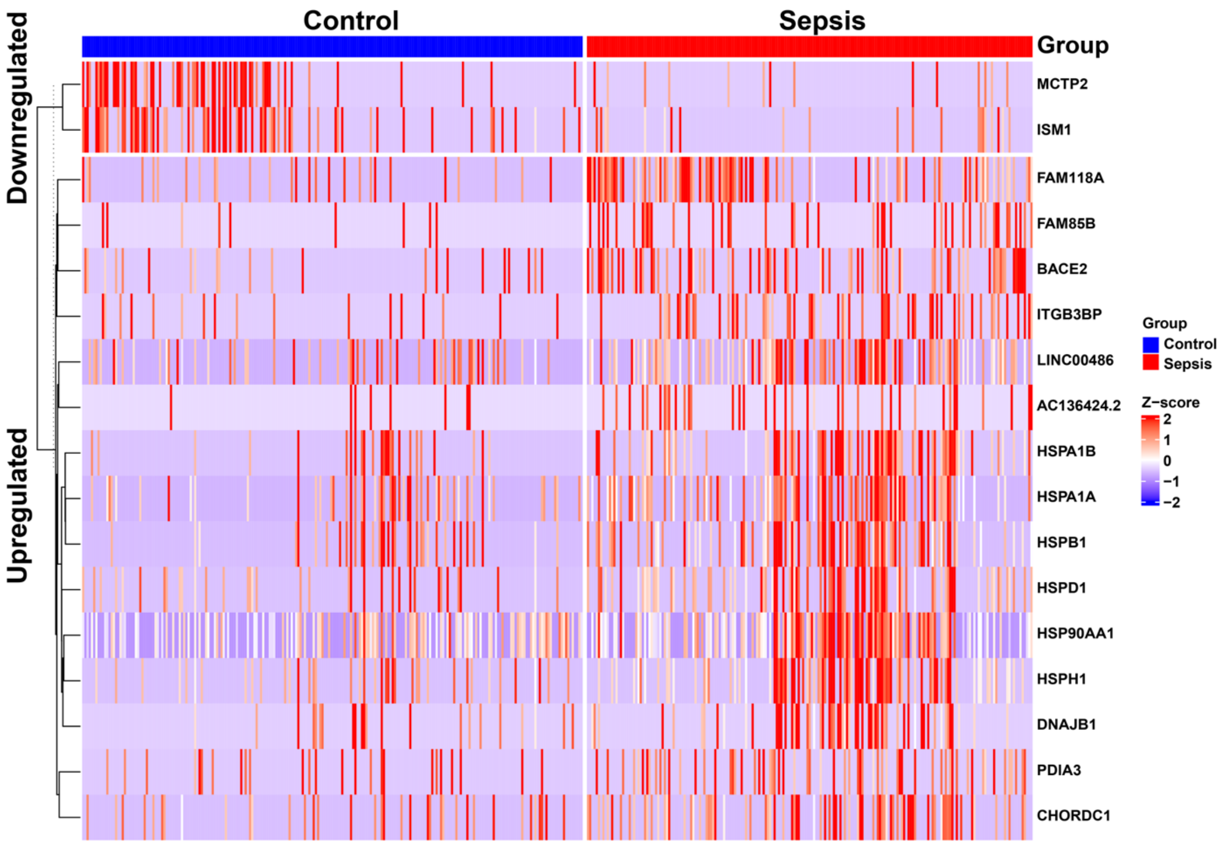

TOP 30 differentially expressed gene in Mural cells

Figure S7. Heatmap showing the top 30 differentially expressed genes (DEGs) in Mural cells when comparing sepsis and control groups. Genes were ranked by average log fold change, with upregulated and downregulated genes included.

Figure S8

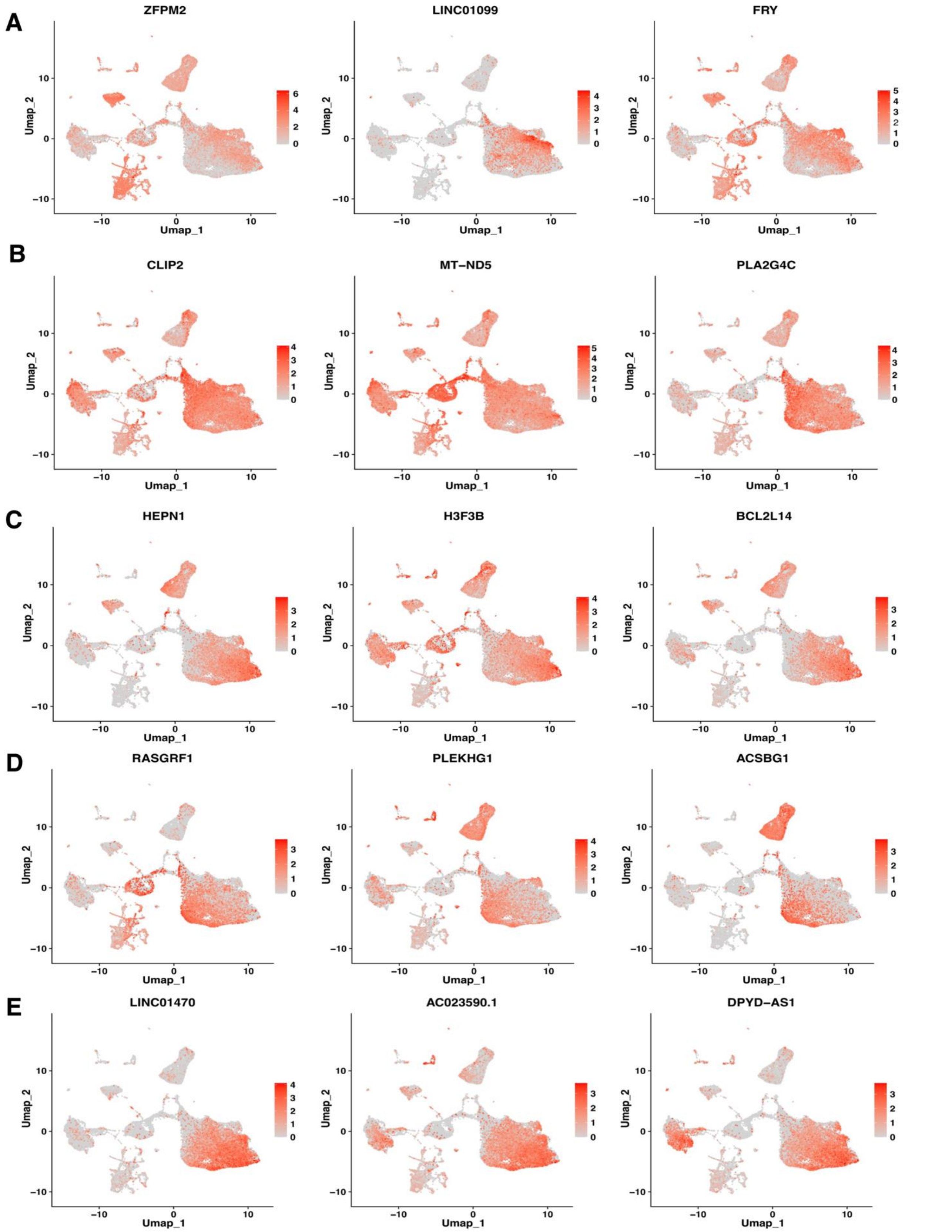
A
ZFPM2
LINC01099
FRY
B
CLIP2
MT−ND5
PLA2G4C
C
HEPN1
H3F3B
BCL2L14
D
RASGRF1
PLEKHG1
ACSBG1
E
LINC01470
AC023590.1
DPYD−AS1
Umap_1
Umap_2

Figure S8. FeaturePlot visualization of representative marker genes for five oligodendrocyte subtypes.

We identified five transcriptionally distinct oligodendrocyte subtypes and visualized representative marker genes for each subtype using Seurat's *FeaturePlot* function. **(A)** Oligodendrocyte 1: expression of **ZFPM2**, **LINC01099**, and **FRY**. **(B)** Oligodendrocyte 2: expression of **CLIP2**, **MT-ND5**, and **PLA2G4C**. **(C)** Oligodendrocyte 3: expression of **HEPN1**, **HEF3B**, and **BCL2L14**. **(D)** Oligodendrocyte 4: expression of **RASGRF1**, **PLEKHG1**, and **ACSBG1**. **(E)** Oligodendrocyte 5: expression of **LINC01470**, **AC023590.1**, and **DPYD-AS1**. Each gene's expression is projected onto the UMAP embedding of all oligodendrocyte cells to illustrate the spatial distribution of subtype-specific markers.

Figure S9

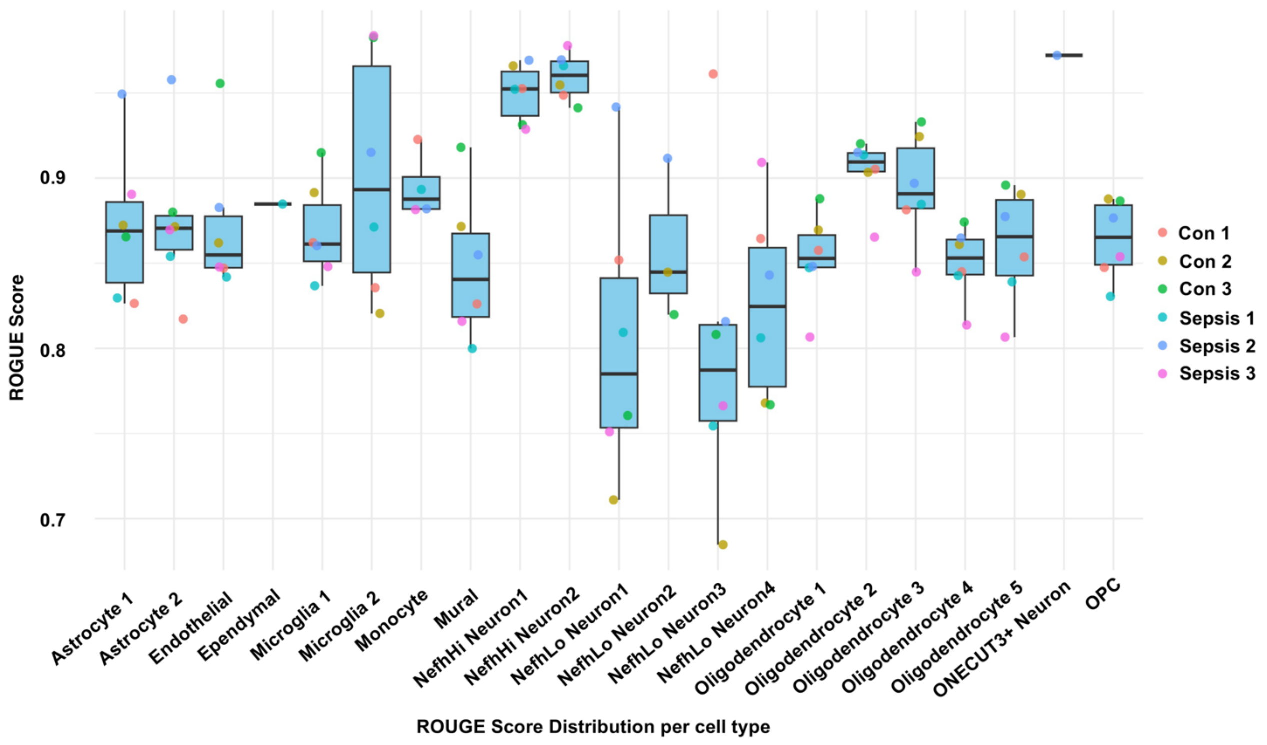

Figure S9. Cluster purity assessment using the ROGUE algorithm.

We performed cluster purity analysis using the ROGUE (Represents Outcome of Gene Expression) R package. ROGUE quantifies the transcriptional homogeneity of each cluster based on entropy measurements of gene expression across individual cells. Higher ROGUE scores indicate greater intra-cluster uniformity. The analysis was applied to all major cell types to evaluate clustering quality in the integrated dataset.

Figure S10

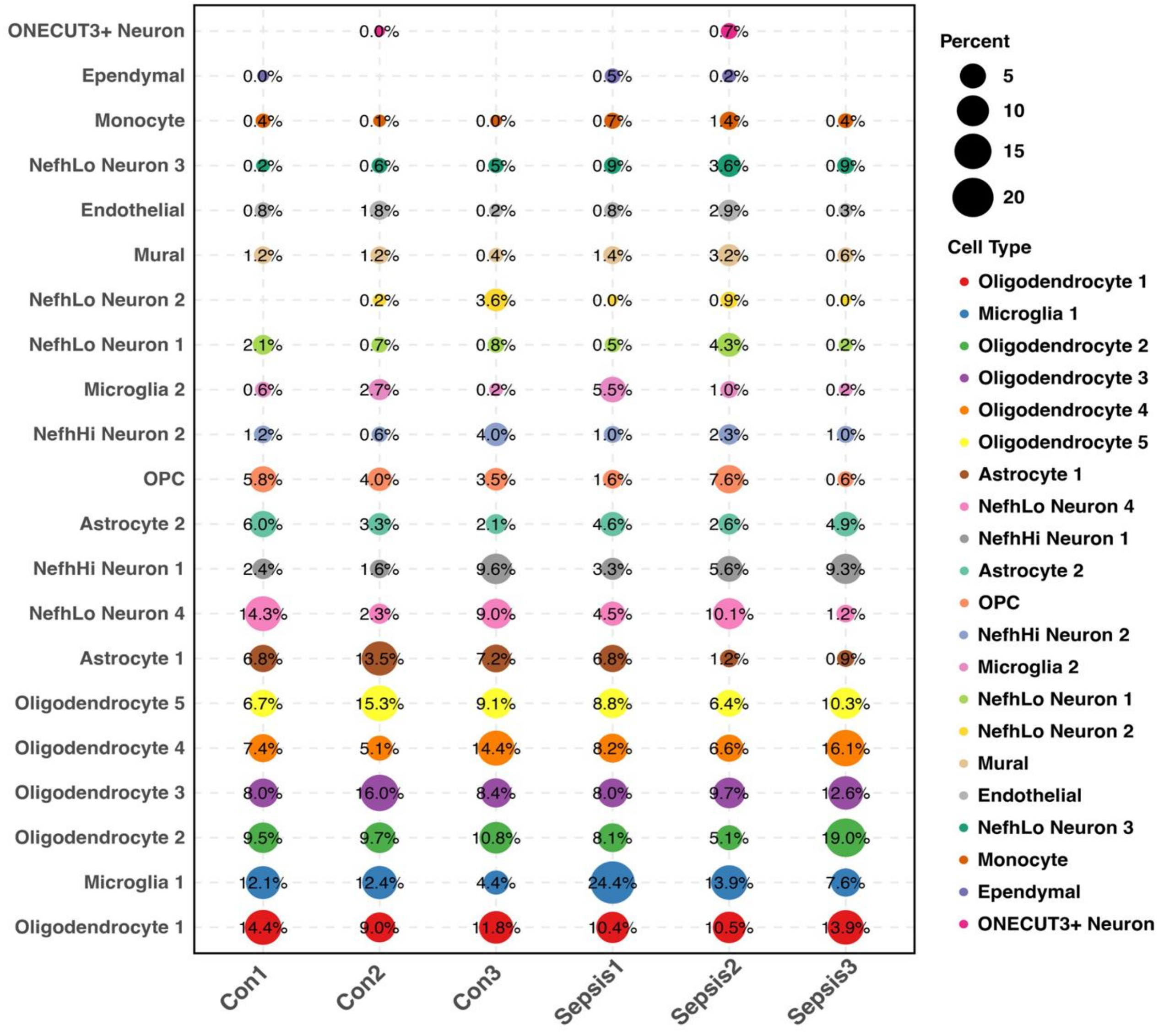

Figure S10. Bubble plot showing the relative proportions of each cell type across individual donors in control and sepsis groups.

We performed a detailed analysis of the proportions of each identified cell type across all six human hippocampal samples, including three controls and three sepsis donors. Each bubble represents the proportion of a given cell type within a specific sample. The size of the bubble reflects the relative abundance of the cell type, and the numeric label inside each bubble indicates the exact percentage value. Samples are grouped by condition (Control vs. Sepsis), allowing visualization of inter-donor variability and sepsis-associated shifts in cellular composition.

Figure S11

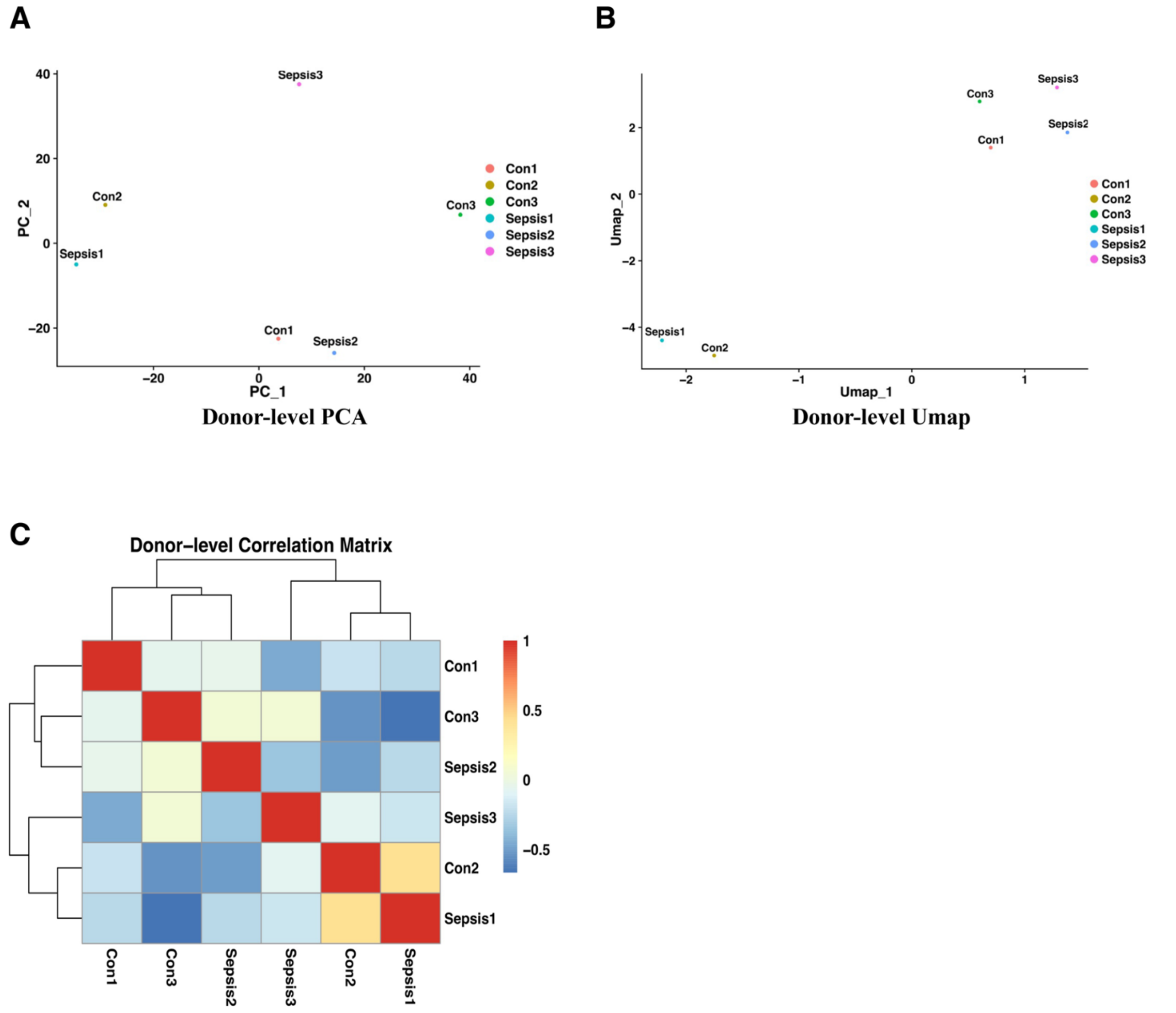

Figure S11. Donor-level pseudo-bulk transcriptomic comparisons.

(A) Principal Component Analysis (PCA) of donor-level pseudo-bulk expression profiles. Gene expression values were averaged across all nuclei per donor to generate a single transcriptomic profile per individual (n = 3 per group). PCA shows partial separation between sepsis and control donors. **(B)** Uniform Manifold Approximation and Projection (UMAP) of the same pseudo-bulked donor-level profiles. Donors from the same condition (sepsis or control) tend to cluster together, supporting the reproducibility of group-specific transcriptomic signatures. **(C)** Pearson correlation matrix heatmap of pseudo-bulk expression profiles. Correlations were computed across all expressed genes for each pair of donors. Higher intra-group correlations were observed compared to inter-group correlations, further indicating consistency within experimental groups.
