# Supplementary material for: Single-nucleus transcriptomics reveals sepsis-related neurovascular dysfunction in the human hippocampus
Source: Front Immunol. 2025 Sep 15;16:1648278. doi: 10.3389/fimmu.2025.1648278 (PMC12477015; doi:10.3389/fimmu.2025.1648278)
Supplement: Supplementary file 2 [file Table1.docx]

**Supplementary Table 1. Literature Sources for Marker Genes of each Cell Type.**

| **Cell Type** | **Representative Marker Genes** | **References（PMID）** |
| --- | --- | --- |
| Endothelial | CLDN5 | 29443965, 35165441; |
| Mural | CSPG4, PDGFRB, ACTA2 | 29443965; |
| Monocyte | CCR2, PTPRC | 28428369, 26060301; |
| Microglia 1 | PTPRC, AIF1, C1QA, CD68 | 30760929, 33257666; |
| Microglia 2 | PTPRC, AIF1, C1QC, CD74 | 30760929, 33257666; |
| OPC | VCAN, CSPG4, OLIG2 | 27284195; |
| Oligodendrocyte 1–5 | MAG, MOG, ERMN, MOBP, MBP, PLP1 | 27284195, 26687838; |
| Astrocyte 1, 2 | GFAP, AQP4, SOX9, ALDH1L1, SLC1A3 | 26687838, 32139688; |
| NefhHi Neuron 1, 2 | MAP2, RBFOX3, NEFM, NEFH, SYN1, SLC17A7 | 27339989, 21562254; |
| NefhLo Neuron 1–4 | MAP2, RBFOX3, NEFM, NEFH, SYN1, SLC17A7 | 27339989, 21562254; |
| Ependymal | FOXJ1, CROCC2 | 29379049; |
| ONECUT3+ Neuron | ONECUT3, ADCYAP1, RBFOX3 | 27716510, 27991900. |
