# Supplementary material for: Single-nucleus transcriptomics reveals sepsis-related neurovascular dysfunction in the human hippocampus
Source: Front Immunol. 2025 Sep 15;16:1648278. doi: 10.3389/fimmu.2025.1648278 (PMC12477015; doi:10.3389/fimmu.2025.1648278)
Supplement: Supplementary file 3 [file Table2.docx]

**Supplementary Table 2. Literature Sources for Inflammatory Genes**

| **Gene set** | **Representative Marker Genes** | **Key References (PMID)** |
| --- | --- | --- |
| **Inflammatory gene** | ACTA2 | 19409525 |
|  | NLRP1 | 32640751, 39443194 |
|  | ALOX5AP | 19126581, 19754386 |
|  | ANXA1 | 26885535, 36306664 |
|  | C1QC | 26410546, 28352669 |
|  | CALCRL | 28352669, 36618798 |
|  | CCL2 | 12851642, 34233864, 28242024 |
|  | CCN1 | 24638890, 20164416, 26099024 |
|  | CD68 | 21268010, 27869795 |
|  | CD74 | 32004754, 27752708, 29476963 |
|  | CD9 | 30356731, 26378766, 19414803 |
|  | CD86 | 15744129, 37918715 |
|  | CEBPA/CEBPG | 30852139, 25591788 |
|  | CH25H | 32229247, 24994901 |
|  | CHI3L1 | 38667293, 38125621 |
|  | CLEC5A | 27795434, 36048544 |
|  | CXCR4 | 30105558 |
|  | CTSL | 9559762 |
|  | CRK | 25621495 |
|  | CYTL1 | 26800213 |
|  | DAB2 | 27748405, 33178208 |
|  | FOS/JUN/JUNB | 26458100 |
|  | FAS | 25655947 |
|  | HLA-B | 18832704 |
|  | HLA‑DRA / B1 / B5 | 22723597，30374345 |
|  | C3, CDKN1A, GADD45A/B | 29247995 |
|  | GPNMB | 34054862, 38782114 |
|  | GFAP | 38216970 |
|  | GPR34 | 39358444 |
|  | IER2, IER5L | 36301664, 38106972 |
|  | S100A10 | 31467414 |
|  | TNFAIP2 | 36084515, 30145807 |
|  | STAT6 | 24416647 |
|  | GBP1 | 35179710 |
|  | RELT | 37893069 |
|  | PDPN | 29269852 |
|  | NUPR1 | 39090667 |
|  | SERPINH1 | 35058967 |
|  | SERPINE1 | 38777211 |
|  | TGFB1 | 30995507 |
|  | MMP2&MMP14 | 24631662，28120021 |
|  | SP1 | 17233566 |
|  | P2RY13 | 35982893 |
|  | TREM2 | 32959884 |
