# Supplementary material for: Single-nucleus transcriptomics reveals sepsis-related neurovascular dysfunction in the human hippocampus
Source: Front Immunol. 2025 Sep 15;16:1648278. doi: 10.3389/fimmu.2025.1648278 (PMC12477015; doi:10.3389/fimmu.2025.1648278)
Supplement: Supplementary file 4 [file Table3.docx]

**Supplementary Table 3. Literature Sources for Blood-Brain Barrier-related Genes**

| **Gene set** | **Representative Marker Genes** | **Key References (PMID)** |
| --- | --- | --- |
| Blood Brain Barrier gene | ADGRG5 | 31256320 |
|  | BIN1 | 30830563 |
|  | CD163 | 38117255 |
|  | CLDN11 | 29984400 |
|  | CLU | 28765369 |
|  | DPP10-AS1 | 33744851 |
|  | ENOX1 | 24247717 |
|  | F13A1 | 26654441 |
|  | FREM2 | 23221805 |
|  | GLUL | 27740595 |
|  | GRIA2 | 27927949 |
|  | LRP8 | 34321020, 40506610 |
|  | MAP1B | 24700609 |
|  | MAPT | 33100967 |
|  | MOG | 36925938 |
|  | MT3 | 21294658 |
|  | NFASC | 17846150 |
|  | NRXN1 / NRXN3 | 36192459 |
|  | NRGN | 39367201 |
|  | P2RY14 | 39100928 |
|  | RHOJ | 34084168 |
|  | S100B | 24194479 |
|  | SHROOM4 | 17009331 |
|  | SPARCL1 | 27581191 |
|  | TXNIP | 28490373 |
|  | VEGFC | 37191285 |
