# Supplementary material for: Single-nucleus transcriptomics reveals sepsis-related neurovascular dysfunction in the human hippocampus
Source: Front Immunol. 2025 Sep 15;16:1648278. doi: 10.3389/fimmu.2025.1648278 (PMC12477015; doi:10.3389/fimmu.2025.1648278)
Supplement: Supplementary file 5 [file Table4.docx]

**Supplementary Table 4. Literature Sources for Gene Sets of M1/M2 polarization.**

| **Gene set** | **Representative Marker Genes** | **Key References (PMID)** |
| --- | --- | --- |
| M1_genes | CD86 | 28928639 |
|  | CD80 | 37762541 |
|  | HLA-DRA/HLA-DRB1 | 28601280, 32678124 |
|  | IL1B | 26446073, 25800044 |
|  | TNF | 25132469, 26446073 |
|  | CCL2/CXCL10 | 33815877, 32848610 |
|  | NOS2 | 30087595, 36982725 |
| M2_genes | MRC1 | 30087595, 32296307 |
|  | ARG1 | 25495532 |
|  | IL10 | 33408474, 37762541 |
|  | TGFB1 | 32224230 |
|  | CCL22 | 32848611 |
|  | TREM2 | 29621548, 40049315 |
|  | P2RY12 | 37762541 |
