# Supplementary material for: Single-nucleus transcriptomics reveals sepsis-related neurovascular dysfunction in the human hippocampus
Source: Front Immunol. 2025 Sep 15;16:1648278. doi: 10.3389/fimmu.2025.1648278 (PMC12477015; doi:10.3389/fimmu.2025.1648278)
Supplement: Supplementary file 6 [file Table5.docx]

|  | Con 1 | Con 2 | Con 3 | Sepsis 1 | Sepsis 2 | Sepsis 3 |
| --- | --- | --- | --- | --- | --- | --- |
| Age | 88 | 82 | 58 | 83 | 78 | 61 |
| Gender | F | F | M | F | F | M |
| PMI | 11.5h | 12.9h | 9h | 12.8h | 7.56h | 13.2h |
| Neurodegenerative disease | none | none | none | none | none | none |

**Supplementary Table 5. Demographic data for human brain donors.**

Con: Control; F: Female; M: Male;PMI: postmortem interval.
